# Peptide-Based Molecular Glues for Targeted Protein Degradation

**DOI:** 10.3390/pharmaceutics18101208

**Published:** 2026-09-23

**Authors:** Walaa S. Aburayan, Ibrahim A. Alradwan, Beshair A. Alsaffar, Othman A. Alhazzaa, Fahad A. Almughem, Abdullah A. Alshehri, Ibtihal S. Alduhaymi, Mohannad M. Fallatah, Meshael Alturki, Hassa A. Alshaya, Essam A. Tawfik

**Affiliations:** 1 Advanced Diagnostics and Therapeutics Institute, Health Sector, King Abdulaziz City for Science and Technology, Riyadh 11442, Saudi Arabia; waburayan@kacst.gov.sa (W.S.A.); abdualshehri@kacst.gov.sa (A.A.A.);; 2 Healthy Aging Research Institute, Health Sector, King Abdulaziz City for Science and Technology, Riyadh 11442, Saudi Arabia; 3 Wellness and Preventative Medicine Institute, Health Sector, King Abdulaziz City for Science and Technology, Riyadh 11442, Saudi Arabia

**Keywords:** targeted protein degradation, peptide-based molecular glues, E3 ubiquitin ligase, peptides, drug discovery

## Abstract

Targeted protein degradation has transformed drug discovery by replacing occupancy-driven inhibition with event-driven removal of disease-causing proteins, opening previously “undruggable” targets, including transcription factors, scaffolding proteins, and intrinsically disordered regions, to therapeutic intervention. Within this landscape, peptide-based molecular glues have emerged as a distinct and underexplored modality, combining the extended, adaptable interaction surfaces of peptides with the mechanistic simplicity of induced-proximity pharmacology. Unlike bifunctional degraders such as PROTACs, which require the concurrent optimization of two linked ligands, peptide molecular glues act through a single engineered interface, stabilizing cooperative ternary complexes between a target protein and an E3 ubiquitin ligase to drive ubiquitination and proteasomal degradation. This review highlights the current understanding of peptide molecular glue biology across five interconnected dimensions: the mechanistic basis of E3 ligase recruitment and cooperative ternary complex formation; rational design strategies spanning sequence selection, structural stabilization, and computational and AI-guided optimization; the structural and biophysical determinants, such as conformational flexibility, interface complementarity, and thermodynamic and kinetic stability, that distinguish productive from non-productive complexes; and emerging therapeutic applications in oncology, hematological malignancy, neurodegeneration, and inflammatory disease, several of which have already reached clinical evaluation. We further examine the comparative advantages of peptide-based architectures, including reduced molecular weight, access to challenging interfaces, and expansion of the addressable E3 ligase repertoire, against persistent translational barriers in proteolytic stability, membrane permeability, immunogenicity, and manufacturability. Taken together, the evidence positions peptide-based molecular glues not as incremental variants of existing degrader platforms, but as a mechanistically distinct therapeutic class capable of substantially widening the druggable proteome.

## 1. Introduction

### 1.1. Overview of Targeted Protein Degradation

The maintenance of protein homeostasis, or proteostasis, is essential for cellular survival and function [1]. Eukaryotic cells regulate protein abundance through tightly coordinated processes that control protein synthesis, folding, trafficking, and degradation [2]. Two major pathways are responsible for protein turnover: the ubiquitin-proteasome system (UPS) and the lysosomal degradation pathway [3]. The UPS selectively removes short-lived, damaged, or misfolded proteins through ubiquitin-dependent recognition and degradation by the 26S proteasome. In contrast, lysosomes mediate the turnover of long-lived proteins, protein aggregates, and intracellular components through autophagy, endocytosis, and phagocytosis [4]. Together, these systems preserve proteome integrity and ensure cellular homeostasis [5].

The therapeutic exploitation of these endogenous degradation pathways has led to the development of targeted protein degradation (TPD), which is one of the most significant advances in modern drug discovery [6]. Unlike conventional pharmacological approaches that suppress protein activity by binding to a specific site, TPD eliminates disease-associated proteins by harnessing the endogenous degradation machinery [7]. This shift from occupancy-driven to event-driven pharmacology has transformed the conceptual framework of therapeutic interventions and expanded opportunities for targeting previously inaccessible proteins [8].

TPD has evolved in several waves, beginning with mechanistic insights into ubiquitin-mediated proteolysis, leading to the development of proteolysis-targeting chimeras (PROTACs), molecular glues, and, most recently, a broader family of induced-proximity technologies [6]. These advances have collectively positioned protein degradation as a flexible therapeutic approach capable of rewiring cellular protein networks rather than merely modulating protein activity [9].

### 1.2. Limitations of Conventional Small-Molecule Inhibitors

Conventional small-molecule inhibitors suffer from fundamental limitations [10], despite their successful use in pharmaceutical development for decades. Their mode of action largely depends on the presence of well-defined binding pockets, catalytic sites, and allosteric regions that accommodate low-molecular-weight ligands [11]. Consequently, only a limited fraction of the human proteome is considered readily druggable using traditional approaches [12]. Many disease-associated proteins function primarily through protein–protein interactions rather than enzymatic activity [13]. Many transcription factors, scaffolding proteins, chromatin regulators, and signaling adaptors have large, flat interaction surfaces that lack appropriate pockets for small-molecule binding [14]. Additional challenges are provided by intrinsically disordered proteins and conformationally dynamic regulatory proteins, whose structural plasticity further limits traditional ligand discovery [15]. Potent inhibitors, when developed, do not necessarily abrogate biological function through target inhibition [16]. The clinical efficacy of targeted therapies is often reduced by residual activity, compensatory signaling pathways, and acquired resistance mechanisms [17]. These limitations have spurred the pursuit of alternative approaches that can directly remove disease-causing proteins rather than transiently suppress their activity [18].

### 1.3. Molecular Glues as an Emerging Class of Degraders

Among the emerging TPD modalities, molecular glues have attracted considerable attention because of their unique mechanism of action [19]. Rather than functioning as inhibitors, molecular glues induce or stabilize protein–protein interactions, promoting the formation of productive complexes that would otherwise be weak, transient, or non-existent [20]. In degradative systems, molecular glues facilitate the proximity between a target protein and an E3 ubiquitin ligase, enabling ubiquitination and subsequent proteasomal degradation, as illustrated in Figure 1 [19]. Unlike bifunctional degraders, molecular glues achieve their activity through the cooperative assembly of protein complexes driven by a single ligand [21]. This mechanism often generates emergent interactions that cannot be predicted from individual binary binding events alone [22]. The clinical success of cereblon-binding immunomodulatory drugs has demonstrated that induced-proximity pharmacology can produce profound therapeutic effects [23]. These discoveries have established molecular glues as a distinct and clinically validated class of degraders, stimulating growing interest in developing new molecular glue architectures [24].

### 1.4. Emerging Role of Peptides in TPD

In parallel with advances in molecular glue pharmacology, peptides have emerged as increasingly attractive therapeutic modalities [25]. Unlike conventional small molecules, peptides can mimic native protein interaction motifs and engage large, structurally complex surfaces with high specificity and affinity [26]. This capability enables the recognition of protein–protein interfaces that are inaccessible to traditional ligands [27]. Peptides have clear advantages due to their modularity and programmability [28]. Sequences can be rationally designed to bind specific structural motifs, recruit degradation machinery, or stabilize productive protein interactions [29]. These features have spurred growing interest in peptide-based degraders and peptide-guided induced-proximity technologies [30]. Peptide stapling, macrocyclization, introduction of non-canonical amino acids, and sophisticated delivery strategies have recently enhanced peptide stability and intracellular activity [31]. Thus, peptides are increasingly viewed not only as biologics but also as versatile molecular scaffolds capable of addressing some of the most challenging targets in drug discovery [25].

### 1.5. Peptide-Based Molecular Glues: An Underexplored Therapeutic Modality

Peptide-based molecular glues represent a convergence of peptide engineering and induced-proximity pharmacology [32]. By combining the interface recognition capabilities of peptides with the mechanistic simplicity of molecular glues, these systems offer a streamlined approach to TPD [24]. Unlike PROTACs, which require two independently optimized ligands connected by a linker, peptide molecular glues can function through a single engineered interaction surface that promotes productive protein proximity and degradation [24]. Their potential is particularly significant for proteins that remain difficult to target using existing degradation technologies [7]. Because peptides can recognize extended interaction surfaces and conformationally dynamic regions, peptide molecular glues may enable the selective degradation of transcription factors, scaffolding proteins, and intrinsically disordered proteins that have historically been considered undruggable [21]. Importantly, peptide molecular glues should not be viewed as peptide versions of existing molecular glues [33]. Rather, they represent a fundamentally distinct strategy that exploits the structural adaptability of peptides to access a broader range of protein interfaces and potentially recruit a wider spectrum of E3 ligases [34]. This expanded recognition capability may significantly broaden the range of degradable targets and create new opportunities for therapeutic interventions [35].

### 1.6. Current Landscape and Knowledge Gaps

Despite their promise, peptide-based molecular glues remain substantially less developed than PROTACs and small-molecule molecular glues [36]. Most current knowledge of induced proximity, ternary complex formation, and degradation mechanisms has been derived from small-molecule systems, and many mechanistic principles may not translate directly to peptide-mediated interactions [26]. However, several key questions remain unresolved. First, the structural determinants that distinguish degradation-competent from non-productive ternary complexes are not yet fully understood [37]. Although cooperativity, geometry, and complex stability are recognized as important factors, predictive design rules remain limited [38]. Second, the relationship between peptide conformational dynamics and degradation efficiency is poorly defined [39]. Unlike conventional inhibitors, peptide molecular glues must simultaneously balance flexibility, specificity, and productive recruitment of proteins [20]. Third, the majority of E3 ligases remain unexplored in peptide-based systems, limiting our understanding of how peptide-mediated recruitment may expand the available degradation toolkit [40]. Addressing these challenges will require integrating structural biology, peptide engineering, computational modeling, quantitative biophysics, and systems-level proteomics. As these disciplines converge, they are expected to transform peptide molecular glues from a promising conceptual framework into a predictive and broadly applicable therapeutic platform.

Here, we review the emerging field of peptide-based molecular glues for TPD. We discuss mechanisms that control E3 ligase recruitment, ternary complex formation, and proteasomal degradation; design strategies; structural and biophysical considerations; therapeutic applications; advantages over existing degrader technologies; current limitations; and prospects. The unique capability of peptide molecular glues to address difficult-to-target protein interfaces and expand the frontiers of degradable proteomes is emphasized.

## 2. Mechanism of Action

### 2.1. Fundamental Principles of Peptide-Based Molecular Glues

Peptide-based molecular glues act via induced proximity mechanisms to enhance or stabilize protein interactions, thereby enabling selective target degradation [24]. Unlike conventional inhibitors that inhibit protein activity by binding to functional sites, peptide molecular glues exert their effects by remodeling protein interaction networks and favoring the formation of productive multiprotein complexes [41]. Thus, their activity is not only dependent on target recognition but also on their ability to direct proteins into beneficial spatial and functional arrangements [42]. A defining feature of this modality is the use of a single engineered peptide interface to mediate protein recruitment [43]. This architecture distinguishes peptide molecular glues from bifunctional degraders and emphasizes cooperative protein assembly, interface complementarity, and ternary complex geometry [44]. Successful degradation depends on a sequence of interconnected molecular events that collectively determine biological activity [45].

It is important to note that this distinction carries direct implications for affinity optimization: unlike bivalent or bifunctional ligands, in which two independently engineered high-affinity interactions are combined, a peptide molecular glue’s binary affinity for either partner is not inherently beneficial. Excessively strong binary engagement can instead stabilize non-productive binary complexes that sequester the peptide or the target protein away from productive ternary assembly, thereby lowering rather than improving degradation efficiency [46].

### 2.2. E3 Ubiquitin Ligase Recruitment

Peptide-mediated degradation is initiated by the recruitment of an E3 ubiquitin ligase [47]. E3 ligases serve as substrate-recognition components of the ubiquitin–proteasome system and determine which proteins are selected for degradation [48]. Peptide molecular glues exploit this machinery by promoting interactions between an E3 ligase and a target protein that would otherwise not occur or would occur only weakly [19].

In this context, the peptide acts as a molecular adaptor that stabilizes the interaction between both partners [25]. Through rational design or directed evolution, peptide sequences can be engineered to engage ligase surfaces while simultaneously supporting the recognition of the target protein [49]. This induced association places the substrate within the ligase’s catalytic reach and provides a foundation for subsequent ubiquitination [50]. An important advantage of peptide-based systems is their potential to expand the range of recruitable E3 ligases [51]. Current degradation technologies rely heavily on a limited subset of ligases, largely because suitable small-molecule ligands are unavailable for most E3 family members [52]. The larger and more adaptable interaction surfaces provided by peptides may enable the engagement of ligases that remain inaccessible to conventional approaches, thereby broadening the scope of TPD [53].

A well-characterized precedent for how a short peptide motif can engage a dedicated recognition domain is the natural Smc/DIABLO N-terminal tetrapeptide (Ala-Val-Pro-Ile-AVPI). Its co-crystal structure with the XIAP BIR3 domain (PDB 1G73) shows that the N-terminal alanine inserts into a narrow hydrophobic pocket and forms four hydrogen bonds and an electrostatic contact that together reproduce the low-micromolar affinity of the full-length Smac protein using only four residues [54]. Although XIAP operates in the apoptotic rather than the ubiquitin-proteasome pathway, this structure established the founding principle later adopted throughout degron-inspired peptide design: a compact, structurally defined tetrapeptide-scale epitope can be sufficient to engage a recognition groove with high affinity, motivating the search for analogous minimal motifs capable of recruiting E3 ubiquitin ligases beyond CRBN and VHL.

### 2.3. Peptide-Mediated Stabilization of Protein–Protein Interactions

Peptide molecular glues not only recruit but also stabilize protein–protein interactions [55]. Many biologically relevant interactions are inherently weak, transient, or highly dynamic [56]. Such features frequently render them resistant to conventional therapeutic interventions. Peptides can overcome this limitation by increasing the interface complementarity and expanding the buried surface area between interacting proteins [57]. Through multiple noncovalent interactions, including hydrogen bonding, electrostatic contacts, hydrophobic interactions, and van der Waals forces, peptide molecular glues transform transient associations into stable and biologically productive assemblies [58]. This mechanism is particularly advantageous for proteins that naturally interact through broad, relatively featureless surfaces. Cyclic and conformationally constrained peptides are particularly effective because their pre-organized structures present interaction motifs in geometries that favor complex stabilization [59]. Consequently, peptide molecular glues can exploit protein interfaces that are often inaccessible to conventional small molecules [60].

### 2.4. Cooperative Ternary Complex Formation

The formation of a cooperative ternary complex represents the central mechanistic event underlying the peptide molecular glue activity [44]. In degradative systems, this complex typically consists of an E3 ligase, a target protein, and the peptide molecular glue. The efficiency of degradation depends on the successful assembly of this multiprotein structure [61]. Importantly, ternary complexes are not simply the sum of two independent binding interactions [62]. Instead, new intermolecular contacts frequently emerge during complex assembly, providing additional stabilization that enhances overall affinity and functionality [56]. This phenomenon distinguishes induced-proximity pharmacology from conventional ligand-based approaches and explains why degradation activity is often not predictable from binary binding measurements alone [20]. Structural studies have shown that productive ternary complexes frequently arise through a stepwise assembly process [63]. Initial peptide engagement with one protein partner facilitates the recruitment of the second partner, generating new interaction surfaces and stabilizing the complete assembly [64]. The resulting complex often exhibits greater stability than that expected from the individual interactions alone [65].

A landmark illustration of this principle is the ternary complex formed by the BET-family degrader MZ1 with the E3 substrate receptor VHL and the second bromodomain of BRD4 (BRD4^AD2^). The co-crystal structure (PDB 5T35) revealed that a phenyl group projecting from the JQ1-derived warhead docks into a hydrophobic pocket formed jointly by VHL and BRD4^AD2^, creating a de novo protein–protein interface, absent from either binary complex, that buries over 2600 Å^2^ of surface area and accounts for the pronounced positive cooperativity driving selective degradation of BRD4 over the nearly identical paralogue BRD3 (Figure 2) [46]. This case shows concretely how neo-interfacial contacts, rather than the summed strength of two binary interactions, dictate whether a ternary assembly becomes degradation-competent, offering a structural template that peptide-based glue design can emulate.

**Figure 2:**
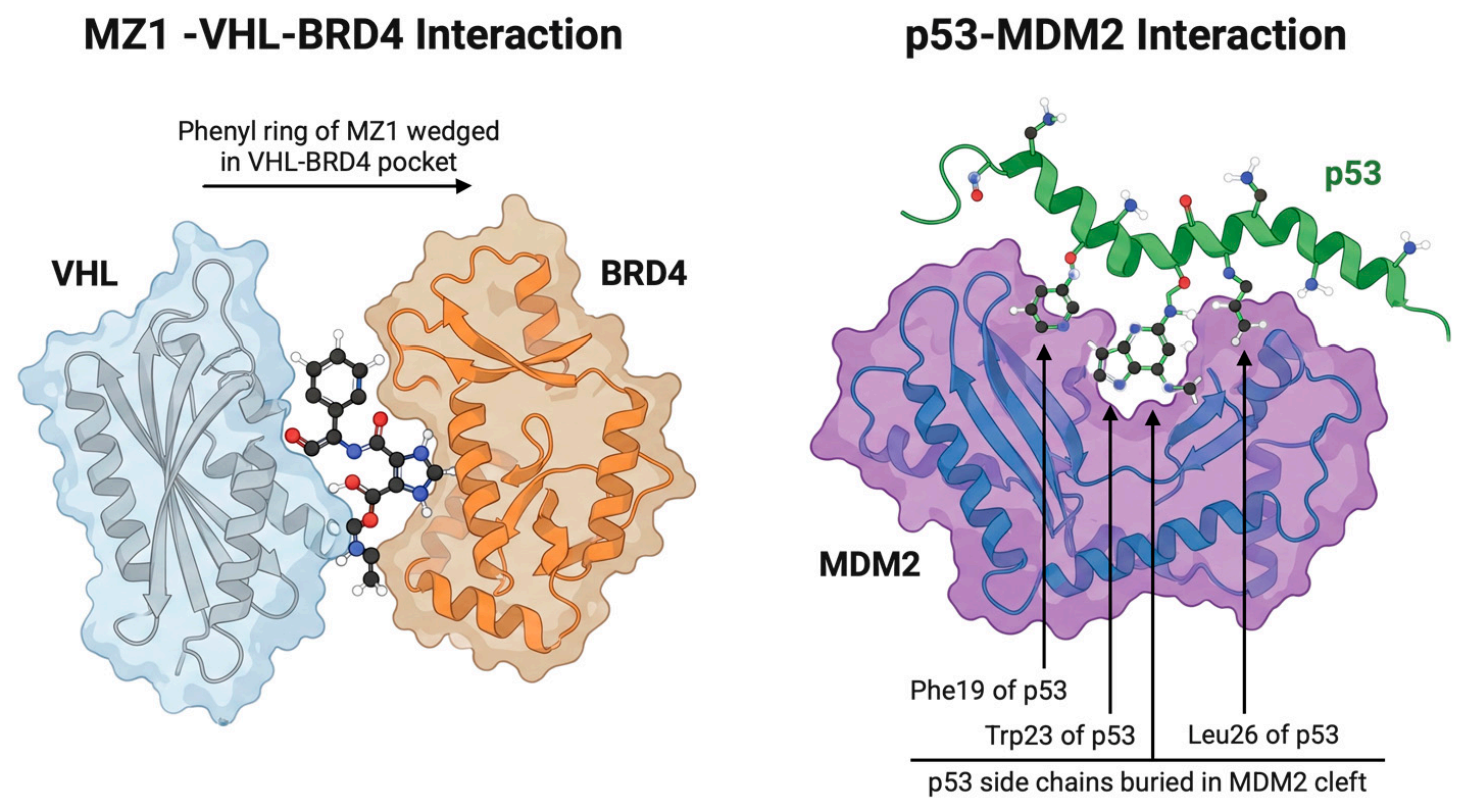
Atomic-resolution basis of cooperativity and interface complementarity in two seminal molecular-glue systems. Created in BioRender. Aburayan, W. (2026) https://BioRender.com/tzo7nko.

### 2.5. Cooperativity as a Driver of Molecular Glue Activity

Cooperativity is widely recognized as one of the most important determinants of molecular glue efficacy [66]. Positive cooperativity occurs when the binding of one interaction partner enhances the recruitment of the second partner, leading to the formation of a more stable and productive ternary complex [67]. In many degradation systems, strong biological activity can be achieved despite relatively modest binary affinities, as most stabilizing interactions emerge only upon ternary assembly [68]. Consequently, degradation efficiency is frequently governed more by cooperativity than by the binding affinity alone. This principle has important implications for molecular design. Optimization strategies that focus exclusively on increasing binary affinity may not necessarily improve degradation outcomes and can be counterproductive [69]. Instead, successful peptide molecular glues must be engineered to maximize productive cooperativity while maintaining a favorable complex geometry and sufficient kinetic stability [70]. Growing evidence suggests that cooperativity may be one of the most reliable predictors of degradation efficiency, highlighting its central role in peptide-mediated induced proximity [30].

### 2.6. Productive Versus Nonproductive Ternary Complexes

Although ternary complex formation is essential, not all complexes lead to degradation of the target protein. A critical distinction exists between productive and non-productive assemblies [37]. Productive complexes position the E3 ligase’s catalytic machinery in proximity to accessible lysine residues or flexible regions of the substrate, thereby enabling efficient ubiquitin transfer [71]. In contrast, nonproductive complexes may exhibit strong binding and high stability but fail to support ubiquitination because of unfavorable spatial organization [72]. This distinction highlights an important challenge in the development of molecular glues. High-affinity binding does not necessarily translate to biological activity. The geometry of the complex, accessibility of ubiquitination sites, and dynamic behavior of the interacting proteins all contribute to determining whether degradation occurs [73]. As a result, the structural characterization of ternary complexes has become increasingly important. Techniques such as X-ray crystallography, cryogenic electron microscopy, hydrogen-deuterium exchange mass spectrometry, and molecular dynamics simulations provide valuable insights into the structural features that distinguish productive degraders from inactive binders [74].

### 2.7. Ubiquitination and Proteasomal Degradation

Once a productive ternary complex is formed, ubiquitination occurs via the canonical E1-E2-E3 cascade. E1 enzymes activate ubiquitin and transfer it to E2 conjugating enzymes, which then transfer it to the substrate via the recruited E3 ligase. This provides a site for the attachment of additional ubiquitins. Cycles of ubiquitin transfer generate a polyubiquitin chain that acts as a molecular signal for recognition and degradation by the proteasome. The polyubiquitylated substrate is then recognized by the 26S proteasome, where it is unfolded, translocated, and degraded to small peptide fragments. This process results in irreversible removal of the target protein, differentiating TPD from classical occupancy-based inhibition. Importantly, the degradation is catalytic, not stoichiometric. A single peptide molecular glue can undergo multiple rounds of degradation, leading to substantial depletion of the target, even without sustained target occupancy. This catalytic behavior is the essence of the potency and durability of degradation-based therapeutic strategies [75,76,77].

### 2.8. Recognition of Flat and Intrinsically Disordered Protein Interfaces

One of the most distinctive advantages of peptide molecular glues is their ability to engage protein surfaces inaccessible to conventional ligands. Many disease-associated proteins function through extended interaction interfaces, intrinsically disordered regions, or conformationally dynamic surfaces that lack well-defined binding pockets [78]. Peptides are particularly well-suited for these targets because they can mimic secondary structural elements and establish extensive intermolecular contacts across broad interfaces [79]. Their adaptable interaction surfaces allow the recognition of topologies that are difficult to address using traditional small molecules [15]. This capability is especially relevant for transcription factors, scaffolding proteins, signaling adaptors, and intrinsically disordered proteins, which collectively constitute a substantial portion of the previously undruggable proteome [80]. By stabilizing specific conformations or imposing local structural order on flexible regions, peptide molecular glues create interaction platforms that support productive degradation [24].

### 2.9. Current Mechanistic Challenges and Knowledge Gaps

While significant progress has been made in the mechanistic understanding of induced-proximity pharmacology, the mechanistic understanding of peptide molecular glues remains limited. The majority of knowledge to date has been gained from small-molecule systems, and it is unclear whether the same principles apply to peptide-mediated degradation. Several unresolved questions continue to limit predictive design. The structural determinants that distinguish productive from non-productive ternary complexes remain incompletely understood [63]. Similarly, the quantitative relationship between cooperativity, complex lifetime, ubiquitination efficiency, and cellular degradation has yet to be fully established [81]. In addition, the influence of peptide conformational dynamics on degradation competence remains largely unexplored [82]. Addressing these questions will require integrating structural biology, quantitative biophysics, proteomics, and computational modeling. Developing predictive mechanistic frameworks that link peptide structures to degradation outcomes is a priority in this field. Peptide-based molecular glues are far more than just simple target binders. Their activity involves a concerted cascade of events, including E3 ligase recruitment, stabilization of protein–protein interactions, cooperative ternary complex formation, ubiquitination, and proteasomal degradation. These processes collectively enable the selective removal of proteins that have long been recalcitrant to therapeutic approaches. As mechanistic understanding progresses, peptide molecular glues are likely to evolve from an emerging concept into a highly programmable degradation platform capable of targeting ever more diverse classes of disease-associated proteins.

## 3. Design Strategies

### 3.1. Design Principles of Peptide-Based Molecular Glues

The design principles of peptide-based molecular glues are fundamentally different from those of conventional inhibitors and bifunctional degraders. Rather than maximizing target occupancy or independently optimizing two binding ligands, peptide molecular glues must be engineered to promote productive protein proximity, stabilize cooperative interactions, and support efficient target degradation [20]. Consequently, successful design relies on integrating structural biology, peptide engineering, biophysical characterization, and computational modeling into a unified framework [83]. Unlike PROTACs, in which linker optimization often plays a central role, peptide molecular glues encode both recognition and functional activity within a single molecular entity [84]. Therefore, their effectiveness depends on the ability to organize protein interfaces into degradation-competent assemblies while maintaining sufficient stability, selectivity, and intracellular exposure [85]. This shift places greater emphasis on ternary complex formation, interface complementarity, and cooperative binding than on binary affinity alone [63].

### 3.2. Peptide Sequence Selection and Interface Engineering

The initial stage in the development of peptide molecular glues is to identify peptide sequences mediating a productive interaction between the target protein and the degradation machinery. Sequence selection is guided by structural information regarding protein interfaces, degron motifs, naturally occurring binding epitopes, and interaction hotspots [86]. Unlike in traditional ligand discovery, the goal is not just to find high-affinity binders. Instead, peptides must be designed to favor protein–protein interactions that cooperatively drive complex assembly. Thus, sequence optimization does not necessarily aim to increase binding strength; rather, it often targets residues that contribute to interfacial complementarity and stabilization. Many successful peptide designs originate from naturally occurring interaction motifs because evolution has already optimized these sequences for protein recognition [87]. Short linear motifs, α-helical segments, β-hairpins, and loop structures frequently serve as starting templates for the engineering of peptide molecular glues. These motifs can be modified to improve their affinity, selectivity, stability, and degradation competence [24]. An important emerging concept is the degron-inspired design. As many E3 ligases naturally recognize short peptide motifs during substrate selection, engineered peptides can exploit similar recognition principles to facilitate ligase recruitment and target engagement [88]. This strategy provides a biologically grounded framework for developing peptide-based degradation systems.

### 3.3. Designing for Cooperativity Rather than Affinity

A major conceptual difference between peptide molecular glues and conventional medicines is their crucial role in cooperativity. Historically, the goal of drug discovery has been to maximize binding affinity, on the premise that stronger engagement with the target will lead to greater biological activity [89]. Induced proximity pharmacology, however, has a different rationale. For peptide molecular glues, degradation efficiency is often determined more by cooperative ternary complex formation than by binary binding affinity [44]. Strong peptide–target interactions alone may be insufficient if they fail to promote the productive assembly of the degradation complex [61]. Conversely, relatively modest binary interactions can produce potent degradation when accompanied by strong positive cooperativity [90]. Therefore, sequence optimization must focus on creating favorable interfacial interactions that emerge during ternary complex formation. This includes maximizing the complementary contacts between the recruited proteins, improving the complex geometry, and stabilizing degradation-competent conformations [24]. Such considerations drive the design process from affinity-centric optimization to the engineering of cooperative molecular assemblies. Importantly, in some cases, too much binary affinity may be detrimental [91]. Very strong interactions can facilitate formation of nonproductive binary complexes and decrease the availability of components required for productive ternary assembly [67]. These findings underscore the importance of balanced optimization strategies that emphasize cooperativity and degradation efficiency over binding strength.

### 3.4. Structural Stabilization Strategies

Structural integrity under physiological conditions is one of the major challenges in peptide-based therapeutic development. Proteases often rapidly degrade linear peptides and can exhibit considerable conformational flexibility, which reduces their binding affinity [92]. Therefore, structural stabilization has become an important aspect in the design of peptide molecular glues. Macrocyclization is one of the most common stabilization strategies. Macrocycles covalently restrict peptide backbones to reduce conformational entropy, improve protease resistance, and often increase binding affinity [93]. Head-to-tail and side-chain cyclizations can also increase structural rigidity while preserving functional recognition motifs [94]. Hydrocarbon stapling is a powerful approach, especially for α-helical peptides. Stapling locks peptides into a helical conformation, thereby increasing proteolytic stability, target recognition, and cellular uptake [95]. The advantages of stapled peptides make them attractive candidates for targeting protein–protein interactions involved in cancer and other diseases [96]. Additional stabilization approaches include N-methylation, incorporation of D-amino acids, β-amino acids, and other non-canonical residues. Such modifications can improve metabolic stability while simultaneously influencing membrane permeability and pharmacokinetics [97]. However, stabilization must be carefully balanced with functional flexibility. Excessive conformational restriction may reduce the adaptability required for cooperative protein assembly and impair the degradation activity [98]. Consequently, successful peptide molecular glue design requires an optimal balance between rigidity and dynamic behavior [99].

The trade-off is not merely a design detail but a central translational constraint: macrocyclization and hydrocarbon stapling improve metabolic stability precisely by restricting the conformational flexibility that peptide molecular glues rely on for adaptive, multi-surface engagement, and the resulting loss of dynamic adaptability can compromise cooperative ternary complex formation. As discussed in Section 7, this stability-flexibility trade-off constitutes one of the principal translational barriers facing peptide-based molecular glues and must be resolved through iterative, degradation-guided optimization rather than stabilization strategies pursued in isolation [96].

### 3.5. Optimizing Cellular Exposure and Pharmacokinetics

Despite their excellent molecular recognition properties, peptides are often limited in therapeutic use due to poor intracellular delivery and adverse pharmacokinetic (PK) properties. Therefore, effective peptide molecular glues need to be designed for both target engagement and sufficient cellular exposure [100]. Several strategies have been developed to improve the permeability and bioavailability of these compounds. N-methylation can reduce the hydrogen-bond donor capacity and promote intramolecular hydrogen bonding, facilitating membrane transport [101]. Macrocyclization can similarly enhance permeability by shielding polar groups and enabling conformational adaptability between aqueous and lipid environments [102]. Lipidation is another commonly used approach. The attachment of hydrophobic moieties can improve membrane association, prolong circulation time, and increase tissue distribution [103]. Likewise, conjugation to cell-penetrating peptides may facilitate intracellular delivery and improve access to cytoplasmic targets [104]. Nanotechnology-based delivery systems are becoming increasingly important. Lipid nanoparticles, polymeric carriers, and targeted delivery platforms provide opportunities to enhance intracellular exposure while protecting peptides from enzymatic degradation [105]. These technologies are likely to play a crucial role in future clinical applications.

Rather than serving as an optional enhancement, nanotechnology-based delivery should be regarded as a prerequisite for in vivo use for most peptide molecular glue candidates: without a delivery platform capable of protecting the peptide from proteolysis and enabling adequate intracellular exposure, even a mechanistically optimized degrader is unlikely to achieve therapeutic activity. This dependency is examined further as a core translational barrier in Section 7 [106].

### 3.6. Computational and Artificial Intelligence-Guided Design

The vast size of the peptide sequence space makes purely experimental discovery inefficient and resource-intensive. Therefore, computational approaches have become essential tools for the development of peptide molecular glues. Structure-based modeling provides a starting point for identifying potential peptide–target protein interaction modes. Docking algorithms, molecular dynamics simulations, and peptide-specific modeling platforms can be used to generate candidate structures and evaluate their compatibility with the desired protein interfaces [107]. These methods help prioritize experimental efforts and reduce the number of peptides that require synthesis and testing [108]. Recently, artificial intelligence (AI) has transformed peptide design. Machine learning models can learn sequence patterns associated with desirable binding properties. At the same time, generative algorithms can produce novel peptide architectures optimized for a given design objective [109]. Diffusion-based approaches and deep learning frameworks have demonstrated particular promise for designing highly specific peptide binders with favorable structural characteristics [110]. Importantly, computational design should not focus solely on affinity predictions. Productive degradation requires favorable cooperativity, ternary-complex geometry, and a sufficiently long complex lifetime. Consequently, future computational workflows must prioritize degradation-relevant parameters over binary binding.

### 3.7. Design–Build–Test–Learn Frameworks

Iterative design–build–test–learn (DBTL) workflows are increasingly relied upon for modern peptide molecular glue discovery. These frameworks integrate computational prediction, experimental validation, and continual optimization into a closed-loop development process [111]. During the design phase, structural and computational analyses generate the candidate sequences. The selected peptides are then synthesized and experimentally evaluated for binding, ternary complex formation, ubiquitination efficiency, and cellular degradation activity [112]. The resulting data are subsequently incorporated into predictive models that guide the next round of design [113]. A major advantage of this approach is its ability to learn from both successful and unsuccessful candidates. Negative results often provide valuable information regarding structural features that impair degradation competence, allowing models to move beyond simple affinity prediction and identify the characteristics associated with true molecular glue activity [24]. As larger degradation datasets become available, DBTL frameworks are expected to accelerate the transition from empirical screening to predictive engineering of peptide molecular glues.

### 3.8. Comparison with Alternative Degrader Architectures

The design principles of peptide molecular glues are fundamentally different from other degradation technologies. Molecular glues stabilize protein interactions with a single ligand [53], but PROTACs rely on bifunctional structures and linker optimization. LYTACs act on extracellular proteins via lysosomal pathways, while AUTACs and related technologies use autophagy-mediated degradation [114]. Peptide molecular glues occupy a special niche in this landscape. They merge the mechanistic simplicity of molecular glues with the interfacial recognition properties of peptides, enabling engagement with protein surfaces that are often inaccessible to both conventional molecular glues and PROTACs. This unique combination provides access to extended protein interfaces, intrinsically disordered regions, and difficult protein–protein interactions of the proteome. Instead of replacing existing degradation modalities, peptide molecular glues should be considered complementary tools that expand the therapeutic toolkit and enable targeting of targets that are still difficult to access with current technologies.

### 3.9. Current Challenges and Future Directions in Design

Despite significant progress, the predictive design of peptide molecular glues remains in its infancy. Most successful systems have been developed through extensive optimization and empirical testing, rather than through fully predictive frameworks. Consequently, many design principles remain incompletely understood. However, several critical questions remain. The structural features that distinguish productive ternary complexes from non-productive ternary complexes remain difficult to predict [115]. As detailed above for the MZ1-VHL-BRD4 case, this balance is becoming tractable on a case-by -case basis, though a general predictive rule remains elusive. Furthermore, the quantitative relationships between cooperativity, complex lifetime, ubiquitination efficiency, and degradation outcomes remain poorly understood [116]. Future advances are likely to emerge through the integration of structural biology, high-throughput screening, machine learning, and quantitative biophysics. As these disciplines converge, design strategies are expected to become increasingly predictive, accelerating the development of peptide molecular glues capable of targeting a broader range of disease-associated proteins. The design of peptide-based molecular glues is a rapidly evolving field at the intersection of peptide engineering, induced-proximity pharmacology, and computational biology. Successful development requires much more than target binding and requires precise control of cooperativity, ternary complex formation, degradation competence, and intracellular exposure. With the increasing predictive power of design tools and mechanistic insight, peptide molecular glues are moving from experimental concepts toward rationally designed therapeutics. These advances set the stage for targeting more difficult proteins and expanding the therapeutic potential of TPD. Figure 3 demonstrates the design principles of peptide molecular glues.

## 4. Structural and Biophysical Considerations

### 4.1. Structural Foundations of Peptide Molecular Glue Activity

The success of peptide-based molecular glues is fundamentally governed by the structural and biophysical principles that determine how proteins recognize, interact with, and stabilize one another. While the design strategies discussed in the previous section focus on generating degradation-competent molecules, structural and biophysical considerations explain why certain peptide-mediated interactions successfully induce degradation, whereas others fail. In contrast to conventional small molecules that bind to pre-existing pockets, peptide molecular glues act at protein–protein interfaces where recognition is typically dynamic, distributed over large surface areas, and highly dependent on conformational adaptability [21]. Thus, understanding the interplay among flexibility, interface complementarity, thermodynamics, and protein dynamics is essential for the rational design of potent peptide degraders.

### 4.2. Conformational Flexibility and Dynamic Recognition

Peptide-mediated molecular recognition is defined by conformational flexibility. Peptides are dynamic ensembles that can sample multiple conformations in solution, unlike rigid small molecules [117]. Such structural flexibility allows them to adapt to a range of protein surfaces and to engage targets that are otherwise inaccessible to traditional ligands. Protein–peptide recognition often occurs through a combination of conformational selection and induced fit mechanisms. In conformational selection, a target protein recognizes and stabilizes a pre-existing peptide conformation. In contrast, the induced fit involves structural rearrangements following the initial binding [118]. In practice, both mechanisms frequently contribute to peptide recognition and collectively shape the formation of productive complexes [119]. This dynamic behavior is particularly important for peptide molecular glues, as they must frequently engage multiple protein surfaces simultaneously. Some flexibility allows for adaptation to different interfaces and promotes the formation of productive ternary assemblies [70]. Too much rigidity may limit adaptability, but too much flexibility may reduce binding specificity and stability [70]. A primary goal of peptide design research is therefore to find an optimal balance between structural organization and conformational freedom. Productive peptide binding often involves transient intermediate states and complex energy landscapes rather than simple lock-and-key interactions, as revealed by molecular dynamics simulations, which have been instrumental in deciphering these processes. These findings underscore the importance of accounting for protein dynamics, rather than static structural information, in the design of peptide molecular glues.

These transient intermediate conformations are increasingly recognized as the principal determinant of complex efficiency, since productive degradation depends on how long the assembly dwells in ubiquitination-competent states rather than on the strength of any single binding event. Accordingly, biophysical characterization of peptide molecular glues should not rely on equilibrium dissociation constants (K_D_) alone; kinetic techniques capable of resolving off-rates and complex residence time, such as surface plasmon resonance, biolayer interferometry, and single-molecule dwell-time analysis, are needed to determine the lifetime of these intermediate and final ternary species and to link it directly to degradation outcomes [120].

### 4.3. Interface Complementarity and Molecular Recognition

The effectiveness of peptide molecular glues depends heavily on the quality of the interface complementarity established between interacting proteins. Productive interactions arise not from a single dominant contact but from the coordinated contribution of multiple intermolecular forces, including hydrogen bonding, electrostatic interactions, hydrophobic contacts, van der Waals interactions, and solvent-mediated effects [58]. Shape complementarity plays an important role in determining the binding specificity. High-affinity interactions often involve significant geometric complementarity between peptide and protein surfaces, facilitating efficient packing and stabilization of the interface [121]. But geometric complementarity alone is not sufficient. Electrostatic interactions often guide the initial molecular recognition, whereas hydrophobic interactions and hydrogen bonds contribute substantially to the stabilization of the final complex [122]. Importantly, peptide molecular glues differ from conventional inhibitors in that they often create new interfaces rather than simply occupying existing binding sites. The resulting neo-interfaces generate emergent interactions that stabilize protein assemblies and contribute to degradation activity [20]. Consequently, successful molecular glue design requires consideration of both the individual binding surfaces and the composite interface formed upon assembly of the ternary complex [20]. Recent studies have also highlighted the contributions of nonclassical interactions, including halogen bonding, π-π stacking, and cation-π interactions [123]. Although less frequently discussed than traditional intermolecular forces, these interactions can significantly influence the binding affinity, specificity, and complex stability.

These forces are not interchangeable in their contribution to binding. Structural and mutagenesis studies of the p53-MDM2 interface showed that a triad of hydrophobic anchor residues on the p53 α-helix, Phe19, Trp23, and Leu26, insert deep into a well-defined hydrophobic cleft on MDM2 and account for the majority of the binding free energy, while surrounding hydrogen bonds and electrostatic contacts refine affinity and specificity rather than driving complex formation (Figure 2) [124]. Building directly on this hierarchy, Bernal and colleagues used hydrocarbon stapling to lock the p53 helix into its bioactive conformation (SAH-p53-8), pre-organizing the Phe19/Trp23/Leu26 triad for MDM2 engagement and thereby reactivating p53-dependent apoptosis in MDM2-overexpressing cancer cells [125]. The lesson for peptide molecular glue design is that identifying and preserving the one or two dominant hydrophobic hotspot contacts is usually more consequential than optimizing the full complement of peripheral polar interactions.

### 4.4. Thermodynamic Determinants of Complex Formation

Stable peptide-mediated protein complexes form under thermodynamic control, with both enthalpic and entropic contributions [126]. However, productive binding occurs only when the overall free-energy change is favorable for complex formation [127]. Historically, strong binding has been attributed to favorable enthalpic interactions, such as hydrogen bonds and electrostatic contacts [128]. However, modern biophysical studies have demonstrated the important role of entropy in many peptide-protein interactions [128]. Entropic gains may arise through solvent displacement, hydrophobic packing, or reduced conformational penalties upon binding [129]. A recurrent phenomenon in peptide recognition is the so-called enthalpy–entropy compensation, in which changes in one energetic component often offset improvements in another. For example, increasing the number of hydrogen bonds may increase enthalpy while decreasing conformational freedom and increasing entropic penalties [130]. Thus, optimization strategies that focus on a single thermodynamic parameter often fail to achieve the desired biological outcome [131]. For peptide molecular glues, thermodynamic optimization should be considered in the wider context of ternary complex formation. The binary interactions must be favorable, but the entire multiprotein assembly must be stabilized for productive degradation. The thermodynamic contributions to these higher-order complexes are an important area of investigation.

### 4.5. Kinetic Stability and Residence Time

Thermodynamic stability decides whether a complex can be formed, whereas kinetic stability decides how long it lasts. There is increasing evidence that residence time is a key control on degradation efficiency [132]. A productive degradation complex must remain assembled for a sufficient duration to permit ubiquitin transfer, polyubiquitin chain formation, and proteasomal recognition [133]. Consequently, the lifetime of the ternary complex may be more important than the equilibrium affinity alone [134]. Short-lived interactions can dissociate before ubiquitination, whereas excessively stable complexes may reduce catalytic turnover and limit degradation efficiency [135]. Surface plasmon resonance, biolayer interferometry, and single-molecule techniques have provided valuable insights into the kinetic behaviors of degrader complexes. These studies increasingly show that degradation activity is more often associated with complex lifetime than binary binding affinity [136]. The growing appreciation for kinetic stability as a design parameter represents an important paradigm shift in degrader development and underscores the need for thorough biophysical characterization beyond conventional affinity measurements.

### 4.6. Role of Solvent and Hydration Networks

Protein–protein interactions occur in a highly hydrated environment, and solvent effects are an important but often overlooked determinant of molecular glue activity. Water molecules can directly influence interface stability by mediating hydrogen-bond networks and by affecting local electrostatic environments [137]. Interfacial water molecules are often involved in recognition processes by bridging peptide and protein surfaces. The loss of ordered water molecules can lead to favorable binding entropy in some instances, whereas in others the preservation of specific hydration networks contributes to stability and specificity [138]. Recently, computational and experimental studies have shown that the dynamics of hydration can play a major role in binding free energies and conformational behavior. These results suggest that solvent effects should be explicitly considered when optimizing peptides, especially in the design of molecules intended to stabilize large protein–protein interfaces [139].

### 4.7. Structural Determinants of Productive Degradation

Not all peptide-mediated interactions result in the degradation of the interacting proteins. Productive degradation requires a precise combination of structural and biophysical factors that extend beyond simple binding affinity [140]. First, the peptide must drive the stable recruitment of both partners. Second, the resulting ternary complex must adopt a geometry that brings the E3 ligase’s catalytic machinery into proximity with the accessible ubiquitination sites on the target protein. Third, the assembly needs to be stable long enough for efficient ubiquitin transfer. Failure to satisfy any of these conditions can result in nonproductive complexes that exhibit strong binding but limited degradation activity [141]. This distinction between binding and degradation competence represents one of the most important concepts in modern TPD. Growing evidence suggests that degradation outcomes are determined by a multidimensional interplay among cooperativity, geometry, dynamics, and kinetics, rather than by affinity alone [136]. In the future, the development of molecular glues will increasingly require integrated structural and biophysical characterization to identify degradation-competent architectures before extensive experimental optimization.

Among these parameters, kinetic residence time has emerged as a particularly strong predictor of outcomes. Comparative SPR analysis of the BET degrader MZ1 bound to the near-identical bromodomains of BRD2, BRD3, and BRD4 showed that a single amino acid difference in BDR3 shortened the ternary-complex dissociative half-life without proportionally reducing equilibrium affinity, and it was this shortened residence time, rather than binding strength, that correlated directly with the reduced cellular degradation of BRD3 relative to its paralogues [120]. This indicates that, when ranking the determinants of degradation efficiency, complex lifetime should generally be weighted above static measures of geometry or affinity alone.

### 4.8. Targeting Intrinsically Disordered Proteins

One of the most promising applications of peptide molecular glues is the targeting of intrinsically disordered proteins (IDPs) and regions (IDRs). These proteins play central roles in transcriptional regulation, signal transduction, phase separation, and cellular organization; however, they remain among the most challenging targets in drug discovery [78]. Unlike folded proteins, IDPs do not have a fixed tertiary structure but rather exist as a dynamic ensemble. This structural heterogeneity makes traditional ligand discovery difficult because there are no stable binding pockets to exploit [142]. Yet, the same flexibility that precludes small-molecule targeting could be advantageous for peptide-mediated recognition [78]. Peptides can recognize preorganized motifs, short interaction hotspots in disordered regions, and transiently populated conformations. Binding often results in local structural organization, transforming dynamic ensembles into competent recognition surfaces for degradation. This phenomenon creates opportunities for targeting proteins that were previously considered undruggable [143]. But it is still a big challenge. Their heterogeneity complicates the structural characterization of IDPs, and predictive design frameworks for disordered targets are still relatively immature. In this area, better integration of structural biology, molecular simulation, and machine learning is needed to fully exploit the therapeutic potential of peptide molecular glues.

### 4.9. Current Knowledge Gaps and Future Directions

Despite substantial advances in our understanding of peptide-protein recognition, many aspects of the biophysics of peptide molecular glues remain poorly understood. Most current knowledge originates from conventional peptide ligands or small-molecule degraders, and it remains unclear whether all established principles translate directly to peptide-mediated induced proximity [24]. But there remain many questions unanswered. What are the structural characteristics of productive and nonproductive ternary complexes? How does conformational dynamics affect degradation efficiency? What thermodynamic and kinetic parameters best predict degradation outcomes? How can these properties be incorporated into predictive design frameworks?

These challenges require ever more sophisticated experimental and computational approaches that can capture the dynamic nature of peptide-mediated recognition. Progress in cryogenic electron microscopy, molecular dynamics simulations, single-molecule biophysics, quantitative proteomics, and AI-based structural prediction will drive this effort. The molecular glue activity of peptides is based on structural and biophysical considerations. The prospects of a peptide for successfully inducing protein degradation are dictated by conformational flexibility, interface complementarity, thermodynamic stability, kinetic behavior, hydration dynamics, and protein disorder, as summarized in Figure 4. With a better understanding of these factors, the field is expected to evolve from empirical optimization to predictive engineering of degradation-competent complexes. Such advances will be crucial for harnessing the full therapeutic potential of peptide-based molecular glues and for extending their application to ever more challenging biological targets.

## 5. Therapeutic Applications

### 5.1. Therapeutic Potential of Peptide-Based Molecular Glues

With the ability to induce selective degradation rather than mere inhibition, peptide-based molecular glues offer unique therapeutic opportunities across a wide range of diseases. These molecules can leverage endogenous protein degradation pathways to target proteins that are not accessible to conventional pharmacological approaches, including transcription factors, scaffolding proteins, and intrinsically disordered proteins [20]. This ability greatly expands the therapeutic landscape and makes peptide molecular glues attractive candidates for diseases driven by previously undruggable targets. Traditional inhibitors are typically occupancy-driven, so they must be bound to the target at all times to be effective. Peptide molecular glues, on the other hand, have an event-driven mechanism. Once degradation is initiated, the target protein is eliminated from the cellular environment, which may result in more durable biological responses and less vulnerability to compensatory signaling mechanisms [20]. These benefits are especially appealing for diseases characterized by an overabundance of proteins, disrupted signaling networks, or reliance on oncogenic proteins.

### 5.2. Applications in Cancer Therapy

Cancer remains the most extensively explored therapeutic area for TPD and currently provides the strongest clinical validation for induced proximity pharmacology. Many oncogenic proteins function through protein–protein interactions, transcriptional regulation, or scaffolding activities rather than enzymatic catalysis, making them difficult to target using conventional small molecules [144]. The clinical success of cereblon-recruiting molecular glues has demonstrated that the selective degradation of transcription factors can produce meaningful therapeutic outcomes. These findings transformed the perception of target druggability and established degradation as a viable strategy for eliminating disease-driving proteins rather than merely suppressing their activity [145]. Peptide-based molecular glues offer several potential advantages in oncology. Their ability to recognize extended protein interfaces may facilitate the degradation of oncogenic proteins that lack traditional ligand-binding pockets [20]. In particular, proteins involved in transcriptional regulation, chromatin remodeling, and signal transduction frequently contain interaction surfaces that are ideally suited for peptide-mediated recognition [146]. Moreover, degradation-based approaches may help overcome several mechanisms of therapeutic resistance. Because the target protein is eliminated rather than inhibited, opportunities for residual signaling and adaptive compensation may be reduced [7]. This feature is particularly relevant for cancers that rapidly develop resistance to occupancy-driven therapies.

### 5.3. Targeting Oncogenic Transcription Factors

Transcription factors are among the most important drivers of cancer progression and remain one of the least tractable target classes in drug discovery. Their biological functions depend primarily on DNA binding and protein–protein interactions, and they generally lack well-defined catalytic pockets suitable for small-molecule inhibition [147]. Peptide molecular glues are uniquely positioned to address this challenge because they mimic native interaction motifs and engage broad recognition surfaces with high specificity. By stabilizing the interactions between transcription factors and degradation machinery, these molecules provide a route toward the selective elimination of proteins that have historically been considered undruggable [21]. In addition to directly degrading transcription factors, peptide molecular glues may also target associated regulatory proteins, coactivators, chromatin remodelers, and transcriptional complexes that collectively drive malignant phenotypes. Such strategies could expand therapeutic opportunities across a wide range of cancers while providing access to previously inaccessible regulatory networks [20].

### 5.4. Hematological Malignancies and Clinical Validation

Among the currently available degradation technologies, hematological malignancies provide the strongest evidence supporting the therapeutic potential of molecular glue pharmacology. Clinical success in multiple myeloma and lymphoma has demonstrated that the targeted degradation of transcriptional regulators can produce durable responses in patients [20]. These results are especially important, as they provide proof of concept for induced proximity therapeutics. Most clinically advanced degraders are based on small-molecule scaffolds, but they offer design principles to guide the development of peptide-based systems [148]. Further illustrating the field’s maturation is the expanding clinical pipeline. Several molecular glue degraders targeting transcription factors and regulatory proteins have entered clinical trials, underscoring the broader potential of degradation-based therapeutic approaches [24]. The clinical momentum establishes a strong foundation for future translational application of peptide molecular glues.

### 5.5. Neurodegenerative Diseases

Neurodegenerative disorders represent another highly promising application area for peptide-based molecular glue. Diseases such as Alzheimer’s disease, Parkinson’s disease, Huntington’s disease, and amyotrophic lateral sclerosis are characterized by the accumulation of toxic proteins that disrupt cellular function and progressively damage neuronal networks [149]. Traditional therapeutic approaches often focus on symptom management and inhibition of downstream pathological processes. In contrast, TPD addresses the underlying cause of the disease by directly removing pathogenic proteins from affected cells [18]. Many proteins implicated in neurodegeneration contain intrinsically disordered regions, aggregation-prone sequences, or extended interaction surfaces that are difficult to target with conventional small molecules. The structural adaptability of peptides provides an opportunity to recognize these challenging targets and facilitate their elimination through endogenous degradation pathways [150]. Proteins such as tau, α-synuclein, mutant huntingtin, and aggregation-prone RNA-binding proteins are particularly attractive candidates for peptide-mediated degradation. Successful removal of these proteins could reduce the aggregate burden, restore proteostasis, and potentially alter disease progression rather than simply alleviating symptoms [151].

### 5.6. Inflammatory and Autoimmune Diseases

Aberrant immune activation and chronic inflammation contribute to numerous pathological conditions, including autoimmune disorders, inflammatory bowel disease, rheumatoid arthritis, systemic lupus erythematosus, and psoriasis. Many proteins that regulate these pathways function through complex interaction networks rather than isolated enzymatic activities. Peptide molecular glues offer an opportunity to selectively remove key signaling components that drive pathological immune responses [20]. By targeting transcription factors, signaling adaptors, cytokine regulators, and other network hubs, degradation-based approaches may achieve broader and more durable modulation of inflammatory pathways than traditional inhibition strategies [20]. This approach is particularly attractive because it allows intervention at the protein abundance level rather than enzymatic activity alone. Elimination of critical regulatory proteins may produce sustained suppression of pathological signaling while potentially reducing the need for continuous pharmacological inhibition [152]. Furthermore, the tissue-specific expression profiles of many immune regulators offer opportunities to develop highly selective degradation strategies that prevent systemic immunosuppression and enhance safety.

### 5.7. Targeting Intrinsically Disordered and Traditionally Undruggable Proteins

One of the most transformative aspects of peptide molecular glues is their potential to address proteins that have historically remained beyond the reach of conventional drug discovery methods. Many disease-associated proteins function through large interaction surfaces, conformationally dynamic regions, or intrinsically disordered domains that lack suitable binding pockets for traditional small molecules [26]. Examples include transcription factors, scaffolding proteins, signaling adaptors, RNA-binding proteins, and numerous regulatory proteins involved in cancer, neurodegeneration, and inflammation. Because peptide molecular glues operate via interface recognition rather than pocket binding, they can exploit structural features inaccessible to most conventional therapeutics [153]. This capability significantly expands the druggable proteome and represents one of the strongest scientific rationales for the continued development of peptide-based degradation technologies. Rather than being constrained by the presence of a ligandable pocket, therapeutic interventions can be guided by biologically relevant interaction surfaces and susceptibility to degradation.

### 5.8. Emerging Applications Beyond Oncology

Although current development efforts are primarily concentrated in oncology and immunology, the potential applications of peptide molecular glues extend far beyond these fields. In infectious diseases, peptide molecular glues may enable the degradation of host factors required for pathogen replication, thereby reducing the selective pressure for the development of resistance [154]. Such approaches could complement traditional antimicrobial therapies and provide alternative strategies for combating drug-resistant pathogens. Metabolic disorders are another promising area of research. Many metabolic diseases involve dysregulated signaling pathways controlled by proteins that are difficult to inhibit directly. Selective degradation of these regulatory proteins can restore physiological homeostasis and provide new therapeutic options [155]. Rare genetic diseases may also benefit from degradation-based strategies. Toxic gain-of-function mutations, dominant-negative proteins, and pathological protein accumulation are common drivers of inherited diseases. The direct elimination of disease-causing proteins offers a potentially powerful strategy for addressing these conditions at their molecular source [35].

### 5.9. Translational Challenges Across Therapeutic Areas

Despite their broad therapeutic potential, several challenges must be addressed before peptide molecular glues can be widely implemented in clinical settings. Efficient intracellular delivery, long-term stability, pharmacokinetic optimization, and selective degradation remain important considerations in all disease areas [156]. Therapeutic requirements also differ widely from indication to indication. For example, neurodegenerative diseases might require efficient penetration of the blood–brain barrier, whereas oncology applications might focus on tumor selectivity and resistance management [157]. These differences highlight the need for disease-specific optimization strategies. Importantly, clinical success will require not only the demonstration of degradation activity but also clear advantages over existing therapy. Therefore, further translational studies are required to evaluate the efficacy, safety, and durability of the response, as well as patient-specific factors that determine degradation outcomes.

The therapeutic applications of peptide-based molecular glues continue to expand as advances in peptide engineering, structural biology, computational design, and delivery technologies improve their practicality. Their unique ability to interact with difficult protein surfaces and remove disease-causing proteins in a novel way makes them a versatile platform with broad relevance across multiple therapeutic areas (Figure 5). Oncology currently has the strongest clinical foundations, but applications are likely to emerge in the future in neurodegeneration, immunology, infectious diseases, metabolic disorders, and rare genetic conditions. As our understanding of disease-specific protein networks deepens and degradation technologies continue to mature, peptide molecular glues may become increasingly important tools for precision medicine and next-generation therapeutic interventions.

## 6. Advantages of Peptide-Based Molecular Glues over Existing Degrader Technologies

### 6.1. Positioning Peptide Molecular Glues Within the TPD Landscape

The rapid expansion of TPD has led to the development of multiple induced-proximity technologies, including PROTACs, molecular glues, lysosome-targeting chimeras (LYTACs), autophagy-targeting chimeras (AUTACs), and related degradation platforms [158]. Each modality offers unique advantages but is associated with distinct limitations. Peptide-based molecular glues represent an emerging class of degraders that combine the interface recognition capabilities of peptides with the mechanistic simplicity of molecular glues [24]. Unlike bifunctional degraders, which require the concurrent optimization of two ligands linked by a linker, peptide molecular glues act via a single engineered interaction surface. This streamlined architecture enables the direct modulation of protein–protein interactions while reducing many of the design challenges associated with more complex degrader systems [24]. Consequently, peptide molecular glues occupy a unique position within the degradation toolbox and may complement existing modalities by targeting proteins that remain difficult to address using current approaches.

### 6.2. Simplified Molecular Architecture

One of the most significant advantages of peptide molecular glues is their simple molecular design. PROTACs require careful optimization of three separate components: a target-binding ligand, an E3 ligase ligand, and a linker connecting the two [53]. Small modifications to any of these elements can substantially influence the degradation efficiency, often necessitating extensive iterative optimization. In contrast, peptide molecular glues encode recognition and functional activity within a single molecule. Rather than relying on linker-mediated proximity, they directly stabilize productive protein interactions through an engineered peptide interface [26]. This simplification reduces the structural complexity and may accelerate lead optimization by decreasing the number of design variables that must be balanced simultaneously [159]. Moreover, the absence of a linker eliminates one of the major challenges in developing degraders. The linker length, composition, and flexibility frequently determine whether a PROTAC forms a productive or nonproductive ternary complex [160]. By avoiding this requirement, peptide molecular glues provide a more direct route to induced proximity pharmacology.

This apparent simplification should not be overstated. Replacing two independently optimizable ligands with a single peptide interface does not necessarily shrink the underlying design space: a peptide engaging an extended protein–protein interface may need to satisfy structural and dynamic constraints across ten or more contact residues simultaneously, spanning both sequence and conformational degrees of freedom, rather than the one or two hotspots typically addressed by small-molecule ligands. This challenge is most acute for intrinsically disordered targets, which lack a fixed binding-competent conformation and instead sample a heterogeneous ensemble from which a peptide must select or induce a productive bound state, coupling folding to binding and adding an entropic and combinatorial dimension absent from rigid-target design [161]. For such targets, the single-interface architecture of a peptide molecular glue does not so much reduce complexity as relocate it, from linker engineering to interface-wide sequence and conformational optimization.

### 6.3. Access to Challenging Protein Interfaces

Perhaps the most important advantage of peptide molecular glues is their ability to engage protein surfaces that are difficult to target using conventional degraders [24]. Most PROTACs rely on small-molecule ligands that bind to specific pockets within the target protein. Consequently, the degradation of proteins lacking suitable ligandable sites remains challenging [24]. Peptides overcome this limitation by recognizing broad and structurally diverse interaction surfaces [79]. Their larger contact areas enable the engagement of protein–protein interfaces, flexible loops, and conformationally dynamic regions that are inaccessible to most small molecules [26]. This capability substantially expands the range of proteins that can be targeted for degradation. Importantly, many disease-relevant proteins, including transcription factors, scaffolding proteins, chromatin regulators, and signaling adaptors, function through extensive interaction surfaces rather than through catalytic domains. Peptide molecular glues are particularly well-suited for these targets because they can mimic native interaction motifs and establish highly specific contacts across large protein interfaces [162].

### 6.4. Targeting Intrinsically Disordered Proteins as a Therapeutic Advantage

Intrinsically disordered proteins (IDPs) represent one of the largest classes of historically undruggable targets [78]. Their lack of a stable tertiary structure makes conventional ligand discovery extremely challenging, and many degradation technologies remain limited by the requirement for well-defined binding sites [78]. Peptide molecular glues offer unique opportunities in this context because they can recognize transiently populated conformations and dynamic interaction motifs within the disordered regions. By inducing local structural organization or stabilizing transient interaction states, peptides can transform conformationally heterogeneous targets into degradation-competent assemblies [21]. This capability distinguishes peptide molecular glues from many existing degrader technologies and may provide access to a substantial portion of the currently inaccessible proteome. Given the important roles of IDPs in cancer, neurodegeneration, and immune regulation, this advantage represents a major therapeutic opportunity.

### 6.5. Enhanced Interface Selectivity

Selectivity remains a critical challenge in TPD because unintended protein elimination can disrupt essential cellular processes [69]. Peptide molecular glues possess several characteristics that may enhance target specificity compared to traditional small molecules [163]. Unlike ligands that interact through relatively localized binding pockets, peptides engage extended interaction surfaces involving multiple recognition elements [24]. This distributed binding mode allows subtle differences in protein topology, electrostatics, and conformational dynamics to contribute to target discrimination [24]. In addition, degradation requires the formation of a productive ternary complex rather than simple target binding. This additional level of selectivity means that even if a peptide interacts weakly with off-target proteins, degradation may not occur unless the complete assembly adopts a geometry compatible with ubiquitination [136]. Consequently, peptide molecular glues may achieve specificity through both molecular recognition and degradation competence [61].

### 6.6. Expanding the E3 Ligase Repertoire

Current degradation technologies rely heavily on a relatively small number of E3 ubiquitin ligases, particularly cereblon and von Hippel-Lindau [164]. This limited repertoire reflects the scarcity of suitable small-molecule ligands capable of recruiting alternative ligases [40]. Peptide molecular glues may help to overcome this bottleneck. Because peptides can engage extended and unconventional protein surfaces, they have the potential to recruit ligases that are inaccessible to conventional small molecules [21]. Expanding the available E3 ligase toolbox would provide several important advantages, including improved tissue specificity, greater flexibility in degrader design, and reduced vulnerability to resistance mechanisms associated with the overreliance on a small number of ligases [53]. Furthermore, different tissues express distinct E3 ligase profiles. The ability to recruit a broader range of ligases could facilitate the development of tissue-selective degradation strategies and improve therapeutic precision [40].

It should be emphasized that this expansion remains a prospective, technology-driven goal rather than an established capability. Recruiting E3 ligases beyond the currently validated set will require dedicated screening innovations, such as high-throughput phage, mRNA, or yeast display selections against diverse ligase surfaces, to systematically identify and validate candidate peptide binders. Until such platforms mature, broadening of the E3 ligase repertoire should be regarded as an ongoing technical frontier rather than a realized advantage of peptide-based molecular glues [165].

### 6.7. Reduced Molecular Weight Compared with Bifunctional Degraders

Although peptides are generally larger than conventional small molecules, peptide molecular glues may still offer advantages over many PROTACs in terms of overall molecular complexity [36]. Bifunctional degraders frequently occupy the beyond-rule-of-five chemical space because they incorporate two ligands connected by a linker, often resulting in molecular weights exceeding 700–1000 Da [36]. In contrast, peptide molecular glues achieve induced proximity without requiring bifunctional architectures [70]. This reduction in structural complexity may facilitate the optimization of pharmacokinetic properties, improve synthetic accessibility, and provide greater flexibility for chemical modification [166]. Importantly, the molecular size alone does not determine the therapeutic performance. However, reducing unnecessary architectural complexity may simplify development and enhance optimization opportunities [167].

### 6.8. Compatibility with Advanced Peptide Engineering

Another major advantage of peptide molecular glues is their compatibility with the rapidly expanding toolbox of peptide-engineering technologies [156]. Advances in peptide chemistry have enabled the development of macrocycles, stapled peptides, constrained peptides, non-canonical amino acids, and stimulus-responsive peptide systems with enhanced stability and functionality [168]. These technologies provide opportunities to optimize pharmacological properties while preserving the interface recognition capabilities that make peptides attractive as molecular glues. Unlike many small-molecule degraders, which are constrained by traditional medicinal chemistry frameworks, peptide molecular glues can exploit a wide range of structural modifications tailored to specific biological requirements [169]. The integration of peptide engineering with computational design and machine-learning-guided optimization is expected to accelerate development further and broaden the scope of accessible targets.

### 6.9. Comparison with Alternative Degradation Technologies

Although peptide molecular glues offer several unique advantages, it is important to recognize that no single degradation modality is universally superior. PROTACs currently have the most mature design framework and the most extensive validation process. Molecular glues have favorable pharmacokinetic properties and clinical success [24]. LYTACs enable the degradation of extracellular proteins, whereas AUTACs exploit autophagy pathways to target larger cellular structures [114]. Peptide molecular glues complement, rather than replace, these technologies. Their greatest strengths lie in targeting challenging protein interfaces, engaging IDPs, and exploiting the versatility of peptide-mediated recognition [20]. Consequently, they are likely to occupy a distinct niche within the broader induced-proximity landscape, particularly for targets that remain difficult to address using existing degrader modalities [24]. Table 1 compares different TPD modalities.

### 6.10. Current Limitations of the Advantage Framework

Although peptide molecular glues offer numerous theoretical and practical advantages, many of these benefits remain to be validated experimentally. Most of the available evidence comes from early-stage studies, and direct comparisons with established degradation technologies remain limited [24]. For example, although peptide-mediated recognition provides access to challenging interfaces, issues related to delivery, stability, and intracellular exposure may offset some of these advantages in therapeutic settings [79]. Likewise, the extent to which broader E3 ligase recruitment can be achieved remains largely unexplored [88]. Figure 6 compares the major TPD modalities.

Consequently, many of the claimed benefits should be considered opportunities rather than realized outcomes. Future comparative studies will be required to delineate the specific contexts in which peptide molecular glues offer meaningful advantages over existing degrader technologies. Peptide-based molecular glues offer several attractive advantages, including simpler molecular architecture, access to difficult protein interfaces, improved selectivity, amenability to advanced peptide engineering, and the possibility to expand the repertoire of recruitable E3 ligases. Most importantly, they provide a strategy for targeting proteins that have historically remained beyond the reach of conventional drug discovery [24]. However, their ultimate value will depend on whether these theoretical advantages translate into practical therapeutic benefits. Further advances in peptide chemistry, structural biology, computational design, and delivery technologies should carve their niche in the TPD landscape. As these developments progress, peptide molecular glues may represent a powerful complementary modality with the potential to expand the conceptual and practical frontiers of protein degradation therapeutics.

## 7. Challenges and Limitations

### 7.1. Translational Challenges in Peptide Molecular Glue Development

Despite their considerable promise, peptide-based molecular glues remain an emerging therapeutic modality and face several scientific, technological, and translational challenges. While their ability to engage difficult protein interfaces offers significant advantages, successful clinical implementation requires overcoming limitations in peptide stability, intracellular delivery, pharmacokinetics, immunogenicity, selectivity, and manufacturability [170]. Importantly, a lot of these challenges are interconnected. Strategies to improve one property may adversely impact others. Increasing structural rigidity may improve proteolytic stability but may also reduce the conformational flexibility required for productive ternary complex formation [171]. Likewise, modifications that enhance membrane permeability may compromise solubility or target recognition [172]. Therefore, a balanced optimization strategy at the development stage that considers the entire degradation process rather than individual molecular properties in isolation is critical for successful development [69].

### 7.2. Proteolytic Instability and Structural Vulnerability

One of the most significant barriers to the development of peptide therapeutics is their susceptibility to proteolytic degradation. Endogenous proteases present in plasma, tissues, and intracellular compartments rapidly recognize and cleave many peptide sequences, resulting in short half-lives and reduced therapeutic exposure [92]. This challenge is particularly important for peptide molecular glues because effective degradation often requires sufficient intracellular persistence to support target engagement, ternary complex formation, ubiquitination, and proteasomal processing [24]. Premature peptide degradation can disrupt multiple stages of the degradation cascade, significantly reducing efficacy [19]. Several stabilization strategies have been developed to address this limitation, including macrocyclization, peptide stapling, N-methylation, incorporation of D-amino acids, and the use of non-canonical residues [94]. While these modifications can substantially improve protease resistance, they introduce additional design complexity and must be carefully optimized to avoid compromising biological activity [173]. A persistent challenge is identifying an appropriate balance between stability and flexibility. Excessive structural rigidification may improve resistance to degradation but can reduce the dynamic adaptability often required for productive protein recognition and cooperative ternary complex assembly [174].

### 7.3. Intracellular Delivery and Membrane Permeability

Efficient intracellular delivery remains one of the most critical obstacles in the development of peptide molecular glues [175]. Most degradation targets reside within the cytoplasm or nucleus, requiring peptides to traverse multiple biological barriers before reaching their intended sites of action [175]. The relatively large size and polar nature of peptides often limit their passive diffusion across membranes [176]. As a result, intracellular concentrations may remain insufficient despite favorable target-binding properties [177]. This issue is particularly problematic for degradation-based therapeutics because successful activity requires the simultaneous engagement of multiple proteins within a specific intracellular environment [178]. Numerous delivery strategies have been investigated to overcome these limitations. Cell-penetrating peptides, lipid conjugates, receptor-targeted systems, and nanoparticle-based carriers have shown promise in enhancing cellular uptake [179]. However, internalization alone does not guarantee therapeutic success [180]. Following uptake, many peptides become trapped within endosomal compartments and fail to reach the cytosol [181]. Endosomal sequestration represents a second major barrier that must be overcome to achieve effective intracellular exposure [182]. The development of efficient endosomal escape technologies remains a key priority in peptide molecular glue research.

### 7.4. Pharmacokinetic and Biodistribution Limitations

In addition to delivery challenges, peptide molecular glues often exhibit unfavorable pharmacokinetic properties. Rapid renal clearance, metabolic instability, and poor oral bioavailability can significantly limit systemic exposure and therapeutic effectiveness [170]. Most peptide therapeutics currently require parenteral administration because gastrointestinal degradation and poor epithelial permeability restrict oral delivery [183]. Although injectable formulations are clinically feasible, they may reduce patient compliance and complicate long-term treatment strategies [184]. Furthermore, achieving adequate tissue distribution remains a challenge for many peptide-based systems. Certain therapeutic applications, particularly those involving solid tumors or central nervous system disorders, require efficient penetration into anatomically protected tissues [185]. Overcoming these barriers will likely require integrating peptide engineering with advanced formulation and delivery technologies [185]. Future progress in long-acting formulations, sustained-release systems, and tissue-targeted delivery platforms may help address these limitations and improve the clinical feasibility of these approaches.

### 7.5. Immunogenicity and Safety Concerns

Although peptides generally exhibit lower immunogenicity than large biologics, immune responses remain an important consideration during therapeutic development [186]. Repeated administration can potentially trigger antibody production, alter pharmacokinetics, or induce unwanted immune activation [187]. The risk of immunogenicity may increase when peptides contain extensive chemical modifications, non-canonical amino acids, or synthetic structural elements that are not commonly encountered in endogenous proteins [186]. Consequently, immunological assessment should be incorporated early in the development process [186]. In addition to peptide-specific concerns, degradation-based therapeutics introduce additional safety considerations. Molecular glues actively reshape protein–protein interaction networks and alter protein abundance, creating the possibility of unintended biological effects [24,188]. Unanticipated degradation of off-target proteins may disrupt essential cellular functions and contribute to toxicity [189]. Therefore, a comprehensive safety evaluation requires characterizing both peptide-related and degradation-related risks across multiple biological contexts.

### 7.6. Selectivity and Off-Target Degradation

Achieving precise target selectivity remains a major challenge for all TPD technologies. Unlike conventional inhibitors, molecular glues do not simply bind to proteins; they actively promote the formation of new protein assemblies [20]. Consequently, unintended interactions may generate unexpected degradation events [90]. Even highly optimized peptides may encounter alternative protein partners within the cellular environment [79]. If these interactions support the formation of a productive ternary complex, off-target ubiquitination and degradation may occur [61]. Such effects can be difficult to predict because they depend not only on binding affinity but also on complex geometry, cooperativity, and intracellular protein abundance [141]. Therefore, advanced proteomic profiling, structural characterization, and systems-level analyses are essential for identifying and minimizing unintended degradation events. The development of predictive computational models may further improve the ability to anticipate off-target effects before clinical evaluation.

### 7.7. Challenges in Ternary-Complex Formation

The ability to form productive ternary complexes is the mechanistic foundation of peptide molecular glue activity. However, achieving this outcome remains challenging. Productive degradation requires the simultaneous optimization of multiple parameters, including affinity, cooperativity, geometry, and complex lifetime [134]. Failure to satisfy any of these requirements can result in nonproductive assemblies that exhibit strong binding but limited degradation activity [24]. Importantly, the relationship between binding and degradation remains poorly understood. Some peptides may stabilize protein interactions without promoting ubiquitination, whereas others may support transient ternary assemblies that fail to persist long enough for efficient degradation [61]. These observations highlight the need for mechanistic assays that directly evaluate degradation competence rather than relying solely on conventional binding measurements [140]. Developing predictive frameworks capable of distinguishing productive from non-productive assemblies remains one of the most important challenges in the field [61].

### 7.8. Challenges Associated with IDPs

Although IDPs are among the most attractive target classes for peptide molecular glues, they pose unique challenges. Their dynamic conformational landscapes create moving targets that are inherently difficult to characterize and manipulate [78]. Unlike structured proteins, IDPs exist as heterogeneous ensembles rather than in stable conformations [190]. Consequently, identifying degradation-competent binding modes often requires extensive structural and computational investigations [191]. Moreover, interactions observed under one set of conditions may not necessarily translate across different cellular environments [192]. The current understanding of peptide-mediated recognition of disordered proteins remains limited, and predictive design principles for these systems are still emerging. Advances in molecular simulation, structural biology, and machine learning are essential for addressing these challenges and fully exploiting the therapeutic potential of disordered targets [193].

### 7.9. Manufacturing and Scalability

Practical considerations are another important barrier to clinical translation. Although peptide synthesis is well established, many peptide molecular glue designs incorporate complex modifications, including cyclization, stapling, non-canonical amino acids, and site-specific conjugations [194]. These modifications can increase manufacturing costs, complicate process development, and introduce challenges for reproducibility and quality control. Maintaining consistent product quality is becoming increasingly important as molecular complexity increases [194]. Furthermore, large-scale production must meet regulatory requirements for purity, stability, and batch-to-batch consistency. Addressing these issues is essential for enabling the widespread clinical adoption of peptide molecular glues [194,195].

### 7.10. Potential Resistance Mechanisms

As with other targeted therapies, resistance is a potential long-term challenge. Although clinical experience with peptide molecular glues remains limited, lessons from PROTACs and small-molecule molecular glues suggest several potential mechanisms of resistance [196]. Alterations in E3 ligase expression, mutations affecting ligase recruitment, changes in target protein abundance, and rewiring of degradation pathways may reduce therapeutic efficacy [53]. Additionally, adaptive cellular responses may compensate for degradation-induced perturbations in protein networks [53]. Understanding these mechanisms is important for designing next-generation degraders that maintain long-term activity and minimize therapeutic resistance.

### 7.11. Need for Next-Generation Stabilization and Delivery Technologies

Many of the current limitations ultimately arise from the intrinsic properties of the peptides themselves. Consequently, continued innovation in stabilization and delivery technologies is essential for future progress. Emerging approaches, such as dynamic covalent constraints, genetically encoded non-canonical amino acids, self-assembling peptide systems, environmentally responsive conformational switches, and programmable nanocarriers, offer exciting opportunities to improve peptide performance [100]. These technologies may enable the development of molecules that combine the recognition capabilities of peptides with the pharmacological properties of conventional small molecules [100]. The convergence of peptide engineering, materials science, and nanotechnology is therefore expected to play a central role in the next generation of peptide molecular glue therapeutics [100].

### 7.12. Current Knowledge Gaps and Future Priorities

Despite this rapid progress, several fundamental questions remain unresolved. The structural determinants that distinguish productive ternary complexes from nonproductive ones remain poorly understood. The quantitative relationships between cooperativity, complex lifetime, ubiquitination efficiency, and degradation outcomes are poorly defined. Similarly, the influence of cellular context on degradation activity requires further investigation. Addressing these gaps will require integrating structural biology, quantitative biophysics, systems proteomics, AI, and advanced cellular models. Such interdisciplinary approaches are expected to transform the development of peptide molecular glues from an empirical process into a more predictive and rational one. The challenges facing peptide-based molecular glues are substantial but not insurmountable, as summarized in Figure 7. Advances in peptide chemistry, computational design, delivery technologies, structural biology, and systems-level characterization are steadily addressing many of the limitations that currently restrict clinical translation [197]. Importantly, the same characteristics that create challenges, including structural flexibility, large interaction surfaces, and dynamic recognition mechanisms, also provide many unique advantages that distinguish peptide molecular glues from other degradation technologies. As mechanistic understanding deepens and enabling technologies continue to mature, the balance between opportunity and limitation is expected to shift increasingly in favor of therapeutic applications [197]. Overcoming these challenges is essential for realizing the full potential of peptide molecular glues and establishing them as a versatile platform for targeting proteins that remain beyond the reach of conventional drug discovery.

## 8. Future Perspectives and Conclusions

### 8.1. The Emergence of Peptide-Based Molecular Glues as a New Therapeutic Paradigm

The field of TPD has evolved from a conceptual framework into a rapidly expanding therapeutic discipline capable of targeting proteins previously considered inaccessible to conventional pharmacological interventions. Peptide-based molecular glues have emerged as a promising and distinctive modality that combines the structural adaptability of peptides with the mechanistic simplicity of induced-proximity pharmacology [19]. Unlike traditional inhibitors, which require continuous target occupancy, peptide molecular glues exploit the endogenous degradation machinery to eliminate disease-associated proteins [90]. This shift from inhibition to protein removal fundamentally alters the therapeutic paradigm and creates opportunities to target proteins that lack conventional ligand-binding pockets [20]. By enabling the selective degradation of transcription factors, scaffolding proteins, signaling adaptors, and IDPs, peptide molecular glues significantly expand the boundaries of the druggable proteome [24]. Although the field remains in its early stages, advances in peptide engineering, structural biology, computational modeling, and degradation biology have laid a strong foundation for future development. These advances suggest that peptide molecular glues may ultimately become a distinct therapeutic platform, rather than simply an extension of existing degradation technologies.

### 8.2. Integration with Nanotechnology and Advanced Delivery Systems

One of the most important directions for future research is the integration of peptide molecular glues with advanced delivery technologies [198]. Despite considerable progress in peptide stabilization, intracellular delivery remains a major barrier to therapeutic translation [170]. Nanotechnology provides a powerful opportunity to overcome these limitations by improving pharmacokinetics, protecting peptides from degradation, and enhancing tissue-specific delivery [199]. A broad range of nanocarrier systems, including lipid nanoparticles, polymeric nanoparticles, dendrimers, exosomes, and biomimetic delivery platforms, are currently being explored for peptide therapeutics [105]. These systems can improve circulation time, increase intracellular exposure, and facilitate delivery to tissues that are otherwise difficult to access [105]. Beyond passive delivery, future nanotechnology platforms may actively contribute to degradation. Stimulus-responsive nanoparticles, multivalent scaffolds, and controlled-release systems can enhance target engagement and improve degradation efficiency [200]. Such approaches have given rise to the emerging concept of Nano-TPD, in which delivery systems become active participants in TPD rather than simple transport vehicles [201]. Therefore, the convergence of peptide molecular glues and nanotechnology is expected to play a central role in overcoming current translational barriers and expanding therapeutic applicability.

### 8.3. AI and Computationally Guided Discovery

AI is increasingly transforming the discovery and optimization of peptide therapeutics. The enormous complexity of peptide sequence space, protein interaction networks, and degradation pathways makes purely experimental discovery inefficient and resource-intensive. Computational approaches provide an opportunity to navigate this complexity more effectively than traditional methods. Recent advances in machine learning, deep learning, and generative modeling have enabled the prediction of peptide structures, protein interfaces, and degradation-relevant interactions with unprecedented accuracy [110]. AI-guided workflows can accelerate candidate identification, prioritize promising sequences, and reduce the experimental screening requirements. Future computational platforms are likely to move beyond simple binding predictions to directly forecast degradation outcomes. By integrating structural biology, proteomics, biophysical measurements, and cellular degradation data, next-generation models may predict cooperativity, ternary complex stability, ubiquitination efficiency, and degradation competence before experimental validation [202]. Importantly, AI may also facilitate the discovery of entirely new molecular glue architectures and the uncovering of previously unrecognized protein interaction networks suitable for degradation-based interventions. As these technologies mature, computationally guided design is expected to become a central component of peptide-molecular glue development.

### 8.4. Personalized Protein Degradation Therapies

The growing availability of patient-specific molecular information creates opportunities for highly personalized degradation strategies. Because degradation efficiency depends on factors such as target abundance, protein conformation, mutation status, and E3 ligase expression, peptide molecular glues are particularly well suited for precision medicine applications [203]. Advances in genomics, transcriptomics, proteomics, and systems biology have enabled increasingly detailed characterization of disease-specific molecular landscapes. Combined with improvements in structural prediction and protein–protein interaction mapping, these technologies may allow peptide degraders to be tailored to individual patients or specific disease subtypes. For example, mutations that alter protein interfaces could create new opportunities for selective degradation [203]. Similarly, tissue-specific E3 ligase expression patterns may enable customized degradation strategies that maximize therapeutic efficacy while minimizing off-target effects [113]. Although such approaches remain largely conceptual, they highlight the long-term potential of peptide molecular glues to move beyond generalized therapies toward truly individualized protein-degradation interventions.

### 8.5. Expanding the E3 Ligase Toolbox

A major limitation of current TPD technologies is their dependence on a relatively small number of E3 ubiquitin ligases [204]. Most clinically advanced degraders rely on cereblon or von Hippel-Lindau, leaving the vast majority of the E3 ligase family unexplored [205]. Peptide molecular glues offer a unique opportunity to address this challenge because peptides can recognize larger and more diverse protein surfaces than conventional small-molecule drugs. This capability may facilitate the recruitment of ligases that currently lack suitable small-molecule ligands and significantly expand the available degradation toolkit [203]. Future efforts to identify novel E3 ligase recruiters, characterize tissue-specific ligase expression patterns, and elucidate ligase-specific degradation mechanisms will be essential to maximize the versatility of peptide molecular glues [40]. Expanding the ligase repertoire may also improve selectivity, reduce resistance, and enable degradation strategies tailored to specific disease contexts [88].

### 8.6. Clinical Translation and Emerging Therapeutic Pipeline

The broader molecular glue field has demonstrated substantial clinical potential, particularly in oncology and immune-mediated diseases. Multiple molecular glue degraders have advanced to clinical trials, validating induced-proximity pharmacology as a viable therapeutic strategy and providing important lessons for future peptide-based systems [20]. As peptide molecular glues mature, successful clinical translation will require a multidisciplinary approach that integrates rational design, structural characterization, mechanistic validation, delivery optimization, and biomarker-guided patient selection [194]. Demonstrating selective target degradation, favorable safety profiles, and reproducible therapeutic benefits will be critical for regulatory approval and clinical adoption. Table 2 lists molecular glue degraders in clinical development.

Beyond efficacy, practical considerations such as scalable manufacturing, formulation development, and regulatory standardization will play important roles in determining the long-term success of peptide molecular glues. Continued collaboration among chemists, structural biologists, computational scientists, clinicians, and regulatory experts is essential to accelerate the translation of laboratory discoveries into clinical applications.

### 8.7. Future Outlook

The future of peptide molecular glues is likely to be shaped by the convergence of several rapidly advancing disciplines. Structural biology provides increasingly detailed views of protein–protein interaction networks. AI is transforming molecular design. Nanotechnology enables sophisticated delivery systems. Systems biology and precision medicine have generated unprecedented insights into disease-specific molecular mechanisms. Together, these advances are expected to transform peptide molecular glues from promising experimental concepts into highly programmable therapeutic platforms. Their ability to engage challenging protein interfaces, manipulate protein interaction networks, and exploit endogenous degradation machinery positions them uniquely in the broader landscape of TPD. Most importantly, peptide molecular glues provide a framework for targeting proteins that have historically remained beyond the reach of conventional therapeutics. As technological and mechanistic barriers continue to diminish, their impact is likely to extend across oncology, neurodegeneration, immunology, infectious diseases, metabolic disorders and rare genetic diseases.

### 8.8. Conclusions

Peptide-based molecular glues represent a convergence of peptide engineering, induced proximity pharmacology, and TPD. By combining the interface recognition capabilities of peptides with the catalytic efficiency of protein degradation, this approach offers a unique strategy for eliminating disease-associated proteins that are inaccessible to conventional therapeutic approaches. Their ability to target complex protein–protein interfaces, intrinsically disordered proteins, and previously undruggable regulatory factors significantly expands the scope of therapeutic interventions. Simultaneously, advances in computational design, structural biology, nanotechnology, and systems-level molecular profiling are rapidly enhancing their feasibility as clinical therapeutics. Although significant scientific and translational challenges remain, the pace of innovation in this field suggests that these barriers are increasingly resolvable. As mechanistic understanding deepens and enabling technologies continue to mature, peptide molecular glues are poised to evolve from an emerging concept into a transformative therapeutic platform that can reshape precision medicine and redefine the boundaries of the druggable proteome.

## Figures and Tables

**Figure 1:**
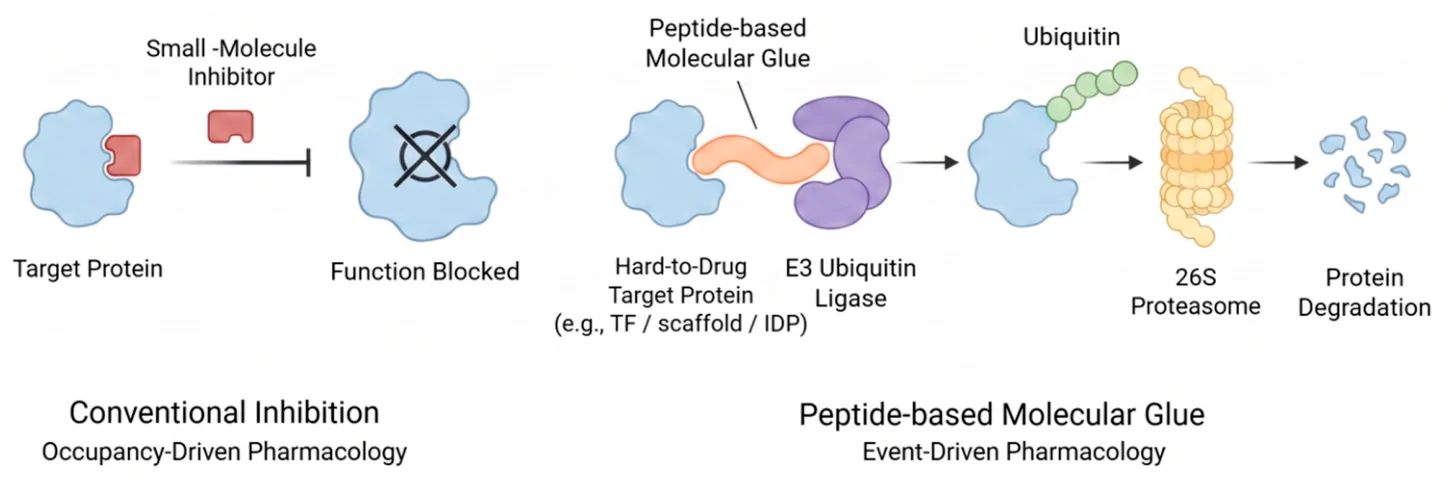
Pharmacological difference between protein inhibition by small-molecule inhibitors and peptide-based molecular glues. Created in BioRender. Mohammed, F. (2026) https://BioRender.com/gr117qq.

**Figure 3:**
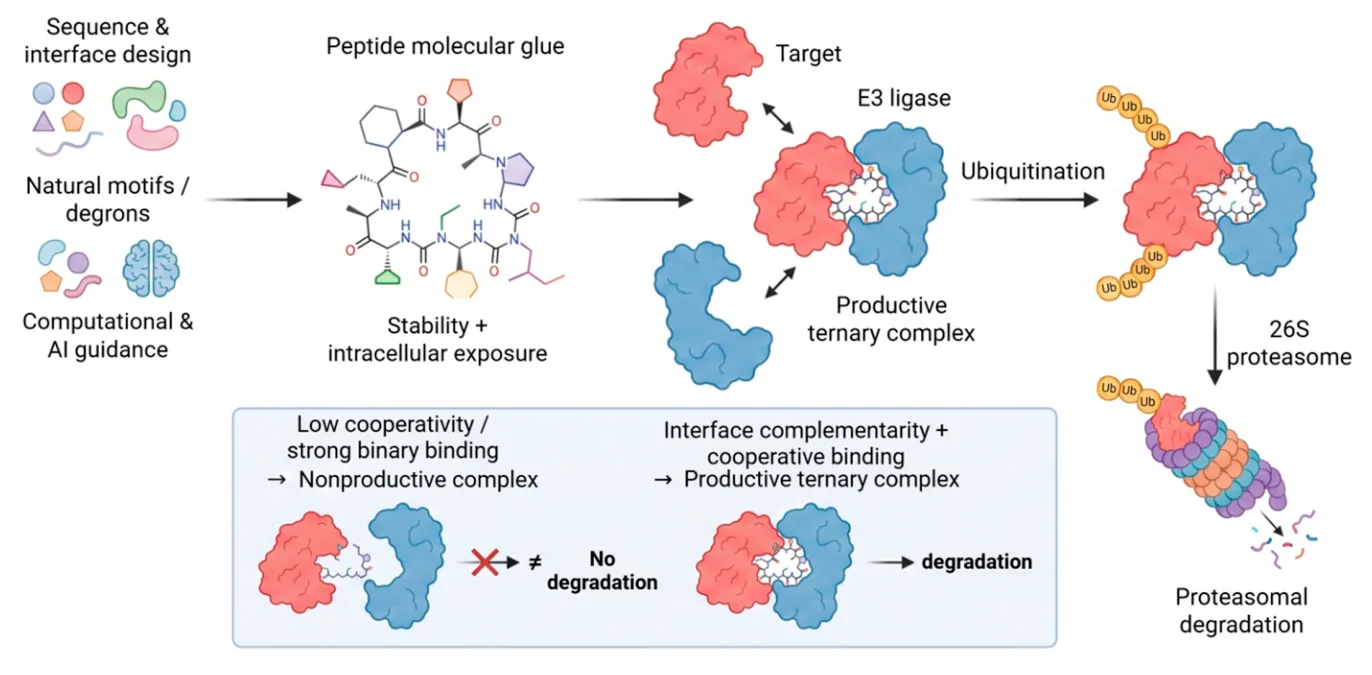
Design principles of peptide-based molecular glues. Created in BioRender. Mohammed, F. (2026) https://BioRender.com/gds9fnh.

**Figure 4:**
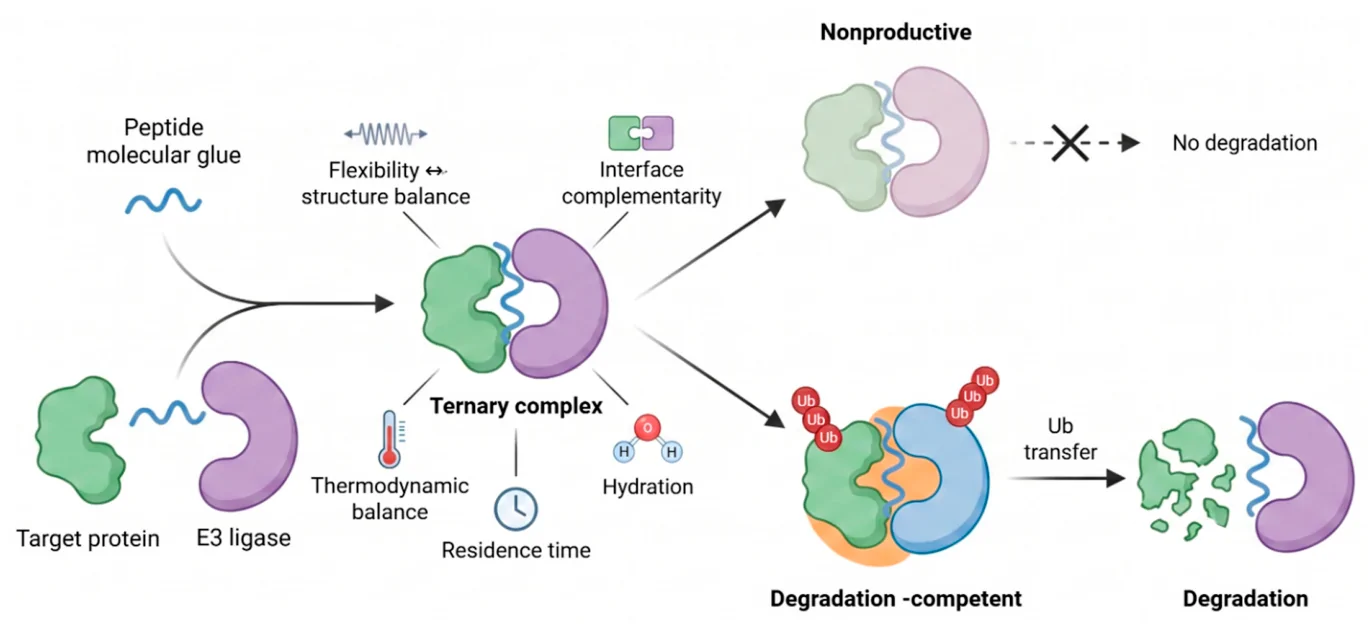
Structural and biophysical considerations of a peptide molecular glue for successfully inducing protein degradation. Created in BioRender. Mohammed, F. (2026) https://BioRender.com/mmxos14.

**Figure 5:**
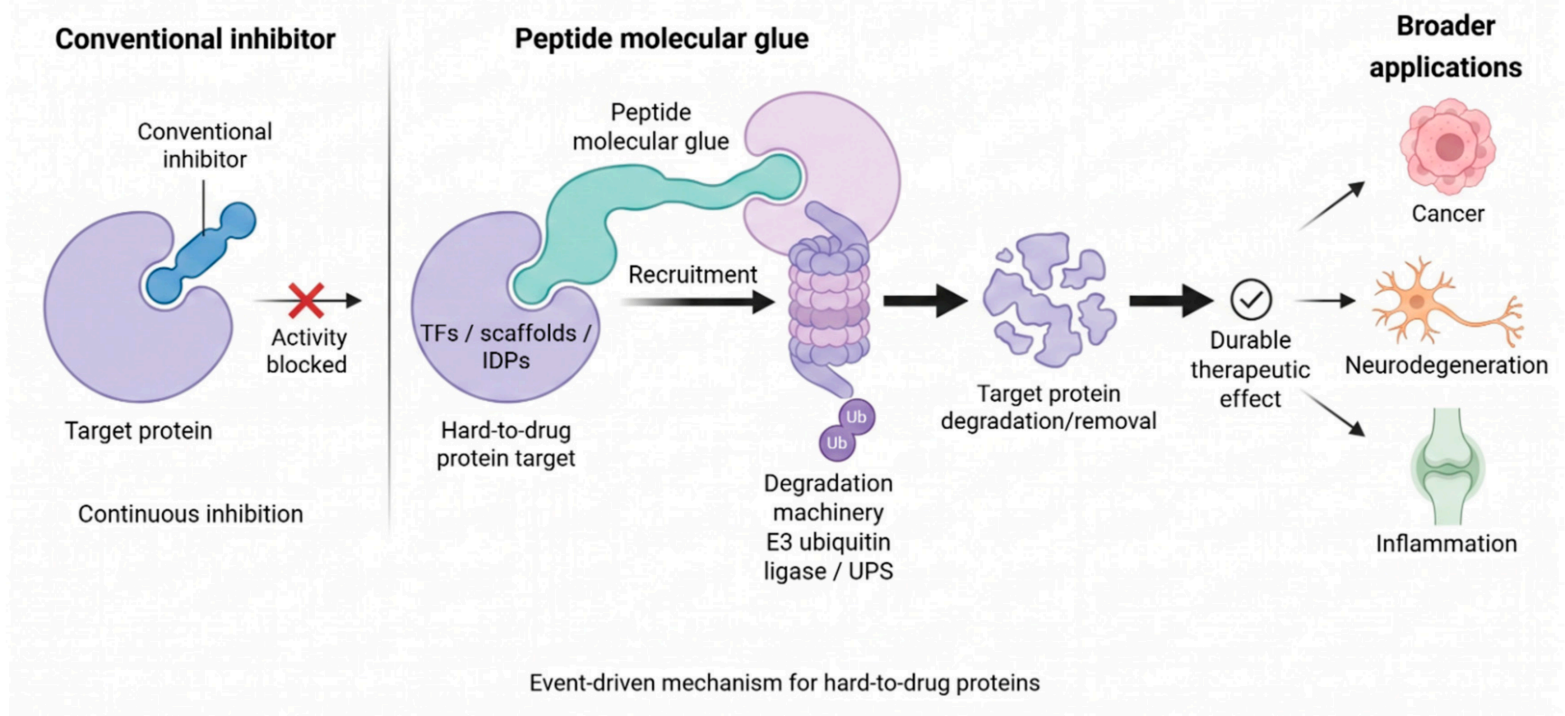
Different potential therapeutic targets of peptide-based molecular glues. Created in BioRender. Mohammed, F. (2026) https://BioRender.com/vev2hfs.

**Figure 6:**
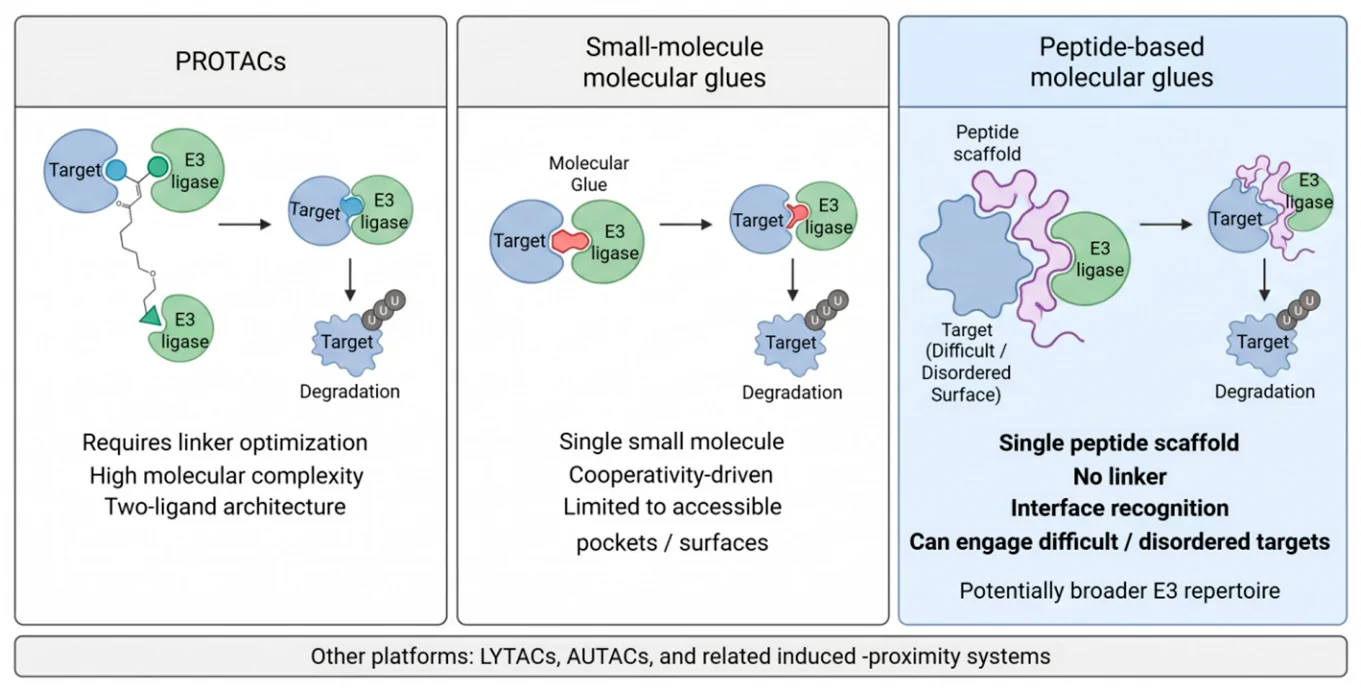
Comparison of peptide-based molecular glues over existing degrader technologies. Created in BioRender. Mohammed, F. (2026) https://BioRender.com/cb4iong.

**Figure 7:**
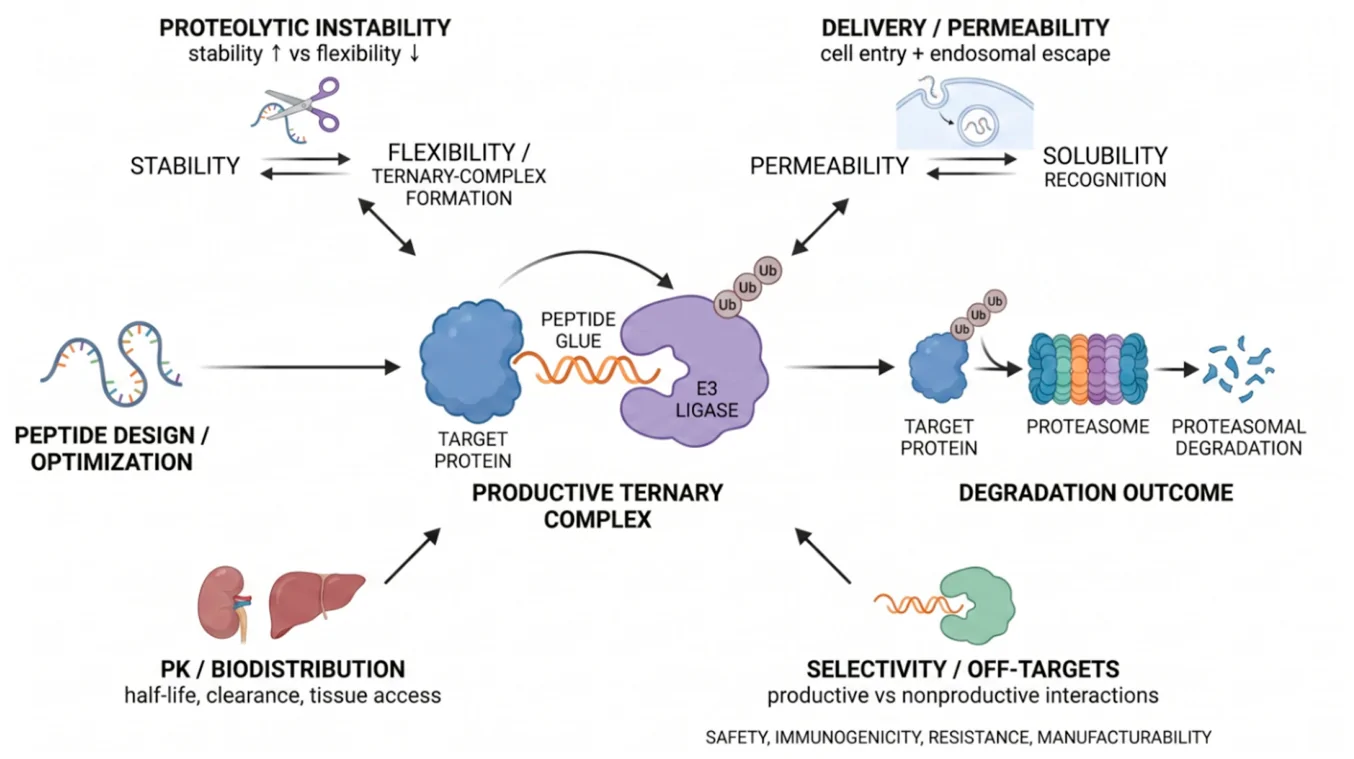
Challenges facing peptide-based molecular glue development and activity. Created in BioRender. Mohammed, F. (2026) https://BioRender.com/5ds8sxl.

**Table 1:** Comparison of major TPD modalities [114].

Feature	PROTACs	Small-Molecule Molecular Glues	Peptide-Based Molecular Glues
**Molecular architecture**	Heterobifunctional molecule consisting of a target ligand, E3 ligase ligand, and linker	Monovalent small molecule	Monovalent peptide scaffold
**Mechanism of action**	Simultaneously binds target protein and E3 ligase to induce proximity	Stabilizes or induces a protein–protein interaction between target and E3 ligase	Stabilizes or promotes target-E3 interactions through an engineered peptide interface
**Requirement for linker**	Yes	No	No
**Molecular complexity**	High	Low	Moderate
**Typical molecular weight**	High (often >700–1000 Da)	Low (<500 Da)	Intermediate, typically dependent on peptide length
**Target engagement strategy**	Occupies two independent binding sites	Creates or stabilizes a neo-interface	Uses peptide-mediated interface recognition and stabilization
**Dependence on ligandable pockets**	Requires ligandable sites on both target and E3 ligase	Requires at least one suitable receptor pocket	Can engage extended protein surfaces without classical pockets
**Ability to target protein–protein interfaces**	Moderate	Moderate	High
**Ability to target IDPs**	Limited	Limited to moderate	High potential
**Surface area available for recognition**	Restricted by small-molecule ligands	Restricted by small-molecule ligands	Large and adaptable interaction surface
**Ternary-complex formation**	Essential and linker-dependent	Essential and cooperativity-driven	Essential and interface-driven
**Role of cooperativity**	Important but often secondary to linker geometry	Primary determinant of activity	Primary determinant of activity
**Access to undruggable targets**	Moderate	Moderate	Potentially extensive
**E3 ligase repertoire**	Currently limited, mainly CRBN and VHL	Currently limited, mainly CRBN and DCAF15	Potentially broader due to peptide-mediated recognition
**Cell permeability**	Often limited	Generally favorable	Frequently challenging
**Proteolytic stability**	High	High	Often requires stabilization strategies
**Oral bioavailability**	Generally poor to moderate	Often favorable	Typically limited
**Delivery requirements**	Moderate	Low	Often requires advanced delivery systems
**Manufacturing complexity**	High	Low	Moderate to high
**Clinical maturity**	Multiple clinical candidates and advanced trials	Multiple approved drugs and clinical candidates	Early-stage and largely preclinical
**Major advantage**	Broad applicability across validated targets	Simple architecture and proven clinical success	Ability to engage challenging protein interfaces and intrinsically disordered targets
**Major limitation**	Large molecular size and linker optimization challenges	Limited discovery strategies and E3 ligase diversity	Stability, delivery, and limited clinical validation

**Table 2:** Selected molecular glue degraders in clinical development [24].

Candidate	Developer	Clinical Stage	Degradation Target(s)	Recruited E3 Ligase	Therapeutic Area
**IKZF1/IKZF3-directed glues**					
**CC-220**	Bristol Myers Squibb	Phase III	IKZF1, IKZF3	CRBN	Multiple myeloma; lymphoma (earlier phase)
**CC-92480**	Bristol Myers Squibb	Phase III	IKZF1, IKZF3	CRBN	Relapsed/refractory multiple myeloma
**CC-99282**	Bristol Myers Squibb	Phase II	IKZF1, IKZF3	CRBN	B-cell lymphoma
**CC-122**	Bristol Myers Squibb	Phase I	IKZF1, IKZF3	CRBN	Lymphoid malignancies
**CFT-7455**	C4 Therapeutics	Phase I	IKZF1, IKZF3	CRBN	Multiple myeloma; non-Hodgkin lymphoma
**KPG-818**	Kangpu Biopharmaceuticals	Phase I	IKZF1, IKZF3	CRBN	Systemic lupus erythematosus; hematologic cancers
**ICP-490**	Innocare Pharma	Phase I	Not publicly disclosed	CRBN	Multiple myeloma; diffuse large B-cell lymphoma
**TQB3820**	Sino Biopharmaceutical	Phase I	IKZF1, IKZF3	CRBN	Multiple myeloma
**Lenalidomide derivative (Starton program)**	Starton Therapeutics	Phase I	IKZF1, IKZF3	CRBN	Multiple myeloma
**GSPT1-targeting glues**					
**CC-90009**	Bristol Myers Squibb	Phase I	GSPT1	CRBN	Acute myeloid leukemia; myelodysplastic syndromes
**MRT-2359**	Monte Rosa Therapeutics	Phase I/II	GSPT1	CRBN	MYC-driven solid tumors
**BTX-1188**	BioTheryX	Phase I	GSPT1; IKZF1/3	CRBN	Hematologic and solid tumors
**Other substrate-directed glues**					
**E7820**	Eisai	Phase II	RBM39	DCAF15	Myeloid malignancies
**NVP-DKY709**	Novartis	Phase I	IKZF2, IKZF4	CRBN	Advanced solid tumors
**KPG-121**	Kangpu Biopharmaceuticals	Phase I	IKZF1/3; CK1α	CRBN	Prostate cancer

## Data Availability

No new data were created or analyzed in this study. Data sharing is not applicable to this article.
